# Patient-Reported Outcome Measures in Cancer Care: An Updated Systematic Review and Meta-Analysis

**DOI:** 10.1001/jamanetworkopen.2024.24793

**Published:** 2024-08-13

**Authors:** Amaris K. Balitsky, Daniel Rayner, Joanne Britto, Anath C. Lionel, Lydia Ginsberg, Wanjae Cho, Ann Mary Wilfred, Huda Sardar, Nathan Cantor, Hira Mian, Mark N. Levine, Gordon H. Guyatt

**Affiliations:** 1 Department of Oncology, McMaster University, Hamilton, Ontario, Canada; 2 Hamilton Health Sciences–Juravinski Hospital and Cancer Centre, Hamilton, Ontario, Canada; 3 Escarpment Cancer Research Institute, McMaster University, Hamilton, Ontario, Canada; 4 Department of Health Research Methods, Evidence, and Impact, McMaster University, Hamilton, Ontario, Canada; 5 Department of Internal Medicine, Thunder Bay Regional Health Sciences Centre, Thunder Bay, Ontario, Canada; 6 Michael G. DeGroote School of Medicine, McMaster University, Hamilton, Ontario, Canada; 7 St George’s University, School of Medicine, Grenada; 8 Arizona College of Osteopathic Medicine, Midwestern University, Glendale; 9 Department of Medicine, McMaster University, Hamilton, Ontario, Canada

## Abstract

**Importance:**

Patient-reported outcome measures (PROMs) come directly from the patient, without clinician interpretation, to provide a patient-centered perspective.

**Objective:**

To understand the association of PROM integration into cancer care with patient-related, therapy-related, and health care utilization outcomes.

**Data Sources:**

Searches included MEDLINE and MEDLINE Epub ahead of print, in-process, and other nonindexed citations; Embase databases (OvidSP); PsychINFO; CENTRAL; and CINAHL from January 1, 2012 to September 26, 2022.

**Study Selection:**

Randomized clinical trials (RCTs) that enrolled adult patients (ages 18 years and older) with active cancer receiving anticancer therapy using a PROM as an intervention.

**Data Extraction and Synthesis:**

Pairs of review authors, using prepiloted forms, independently extracted trial characteristics, disease characteristics, and intervention details. The Preferred Reporting Items for Systematic Reviews and Meta-Analyses reporting guideline was followed. Random-effects analyses were conducted.

**Main Outcomes and Measures:**

Overall mortality, health-related quality of life (HRQoL) measures, and hospital utilization outcomes.

**Results:**

From 1996 to 2022, 45 RCTs including 13 661 participants addressed the association of PROMs with outcomes considered important to patients. The addition of a PROM likely reduced the risk of overall mortality (HR, 0.84; 95% CI, 0.72-0.98; moderate certainty), improved HRQoL (range 0-100) at 12 weeks (mean difference [MD], 2.45; 95% CI, 0.42-4.48; moderate certainty). Improvements of HRQoL at 24 weeks were not significant (MD, 1.87; 95% CI, −1.21 to 4.96; low certainty). There was no association between the addition of a PROM and HRQoL at 48 weeks. The addition of a PROM was not associated with reduced ED visits (OR, 0.74; 95% CI, 0.54-1.02; low certainty) or hospital admissions (OR, 0.86; 95% CI, 0.73-1.02; low certainty).

**Conclusion and Relevance:**

The findings of this study suggest that the integration of PROMs into cancer care may improve overall survival and quality of life.

## Key Points

**Question:**

How does the integration of patient-reported outcome measures (PROMs) affect outcomes of cancer care?

**Findings:**

In this update to a systematic review and meta-analysis of 45 randomized clinical trials examining the use of PROMs for patients receiving anticancer treatment, the integration of PROMs into cancer care likely improved overall survival and HRQoL with moderate certainty. Results for reductions in emergency department visits and hospitalizations were not significant.

**Meaning:**

These results suggest that integrating the patient perspective into cancer care can improve patient outcomes and health resource utilization.

## Teaser

This systematic review and meta-analysis is an update to a previous review of randomized clinical trials examining the association of patient-reported outcome measures used in cancer treatment with overall mortality risk, health-related quality of life, and hospital utilization.

## Introduction

Symptoms, many of which go largely undetected by clinicians,^1,2,3,4^ are common among individuals with cancer.^5^ Even in a tightly controlled clinical trial comparing physician and patient reporting of symptoms, physician reporting was neither sensitive nor specific in detecting common chemotherapy toxic effects.^4^ In addition, clinician-to-clinician agreement when reporting symptoms is moderate at best.^6^ The discrepancy between clinician-reported and patient-reported outcomes suggests that accurate assessment of symptoms and consequent health-related quality of life (HRQoL) requires direct measurement from patients.

Patient-reported outcome measures (PROMs) are measures of symptom burden and HRQoL that come directly from the patient, without clinician interpretation. PROMs can be the intervention and/or the outcome in a trial. In this study, our focus is on the integration of PROMs into oncology care as the intervention.

Possibly due to differences in choice of PROM, population diversity, different selected outcomes, and the different methodologies, previous systematic reviews measuring the association of PROMs with the quality of care across different disease populations have proved inconclusive.^7,8,9,10,11,12,13^ A previous systematic review published in 2014^14^ included 26 studies (randomized clinical trials [RCTs] and non-RCTs) that focused on a PROM as an intervention in cancer care. Authors did not perform a meta-analysis due to the variability in previously noted factors.

Since 2014, the impact of PROMs has come to the forefront of cancer care. The integration of PROMs into cancer care can improve HRQoL and survival, which is potentially attributable to improved symptom management and tolerance of treatment regimens.^15,16^ Given the potential survival benefit of including PROMs into oncology care, we performed an updated systematic review addressing the impact of integrating PROMs into oncology care for patients with cancer undergoing active therapy, focusing not only on survival but also on other patient-valued outcomes, including HRQoL and measures of health care resource utilization like number of emergency department (ED) visits and hospital admissions.

## Methods

We followed the guidelines of the Preferred Reporting Items for Systematic Reviews and Meta-Analyses (PRISMA) reporting guideline. The systematic review was submitted to the International Prospective Registry of Systematic Reviews (PROSPERO) (ID266577).

### Study Selection and Search Strategy

We began by running the search from a previous systematic review published in 2014 (eAppendices 1 and 2 in Supplement 1).^14^ Twenty of the 26 articles from the previous search were RCTs and included in our full-text eligibility evaluation. An experienced information specialist then conducted a comprehensive search in MEDLINE and MEDLINE Epub ahead of print, in-process, and other nonindexed citations; Embase databases (OvidSP); PsycINFO; CENTRAL; and CINAHL from 2012 to September 26, 2022. There were no language or publication status restrictions. To identify other potentially relevant trials, we reviewed reference lists of included trials and relevant review articles.

We included RCTs that enrolled adult patients (ages 18 years or older) with active cancer and receiving anticancer therapy. The intervention was the administration of a PROM compared with standard care without PROM administration. In the intervention group, the results of the PROM had to be shared with the patient’s health care professional. We excluded studies that included survivors of cancer (ie, not on cancer-directed therapy) or included PROMs only as an outcome measure.

Pairs of review authors (J.B., L.G., W.C., N.H., A.W., H.S., N.C., and A.L.) independently screened titles and abstracts for possible inclusion. The team of review authors conducted full-text review of any possibly relevant trials. Review authors resolved discrepancies through adjudication (A.B.).

### Outcomes Collected

We categorized outcomes used to evaluate PROMs as an intervention into 3 categories: patient-reported, clinician-reported, and health care utilization. Patient-reported outcomes included: HRQoL measures, symptom burden measures, and psychological measures. Clinician-reported outcomes included mortality, therapy completion, and therapy complications. Health care utilization outcomes included number of unscheduled clinic visits, number of hospital admissions, and number of emergency department visits.

### Data Extraction and Quality Assessment

Pairs of review authors, using prepiloted forms, independently extracted the following data: trial characteristics, including study design, country, trial setting (eg, clinic, hospital); disease characteristics such as type of cancer and stage of cancer; and intervention details, including type of PROM, timing of administration, and method of administration (eg, paper or electronic). Pairs of review authors independently assessed all eligible studies for their risk of bias using the Cochrane RoB 2.0 tool.^17^ Overall risk of bias for each trial was defined as high risk of bias if there were some concerns in 2 or more domains. Certainty of pooled effect estimates for each outcome were assessed using Grading of Recommendations Assessment, Development and Evaluation (GRADE) methodology.^18,19,20,21^ We rated certainty in a nonzero effect.

### Statistical Analysis

A meta-analysis was performed for each outcome included in at least 2 studies. Results were pooled in DerSimonian-Laird random-effects meta-analyses using the inverse variance method. Dichotomous outcome data were expressed as odds ratios (ORs) and 95% CIs and continuous outcomes were expressed as mean differences (MD). We assessed statistical heterogeneity using a combination of visual inspection of the forest plots along with consideration of the χ^2^ test and the *I^2^* statistic.^22^ The STATA SE version 18 (Stata Inc) metan function provided the software for all statistical analyses.

To explore the impact of including trials with high risk of bias, we removed studies with overall high risk of bias and repeated the meta-analysis without those studies. We conducted a test of interaction between the results of low and high risk of bias groups. The threshold for significance was *P* < .10; if results were significant, we applied ICEMAN (Instrument to Assess the Credibility of Effect Modification Analyses) criteria.^23^

## Results

### Study Selection

**Figure 1.**
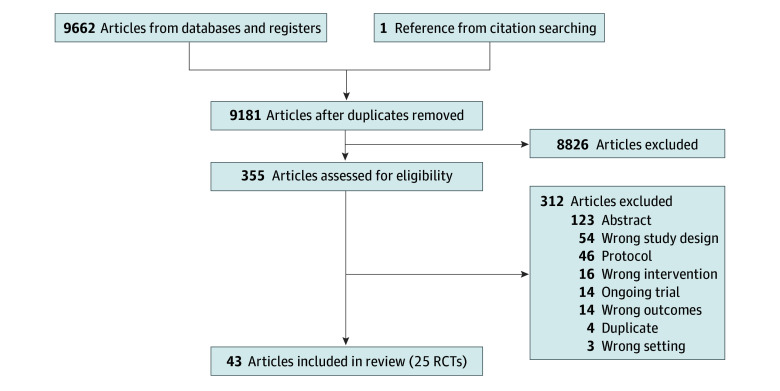
PRISMA Diagram of Article Selection for Updated Review The original meta-analysis included randomized clinical trials (RCTs) and observational trials. We combined the 20 RCTs from the original review with the 25 RCTs from the updated search.

We retrieved 9662 citations, of which 482 were duplicates (Figure 1). One additional study, found in a reference list review, proved eligible. The initial search included RCTs and observational trials. Given that there was a sufficient number of RCTs, we limited inclusion to RCTs only. There were 45 RCTs, 20 from the original search and 25 from the new search.^16,24,25,26,27,28,29,30,31,32,33,34,35,36,37,38,39,40,41,42,43,44,45,46,47,48,49,50,51,52,53,54,55,56,57,58,59,60,61,62,63,64,65,66,67,68^

### Study and Patient Characteristics

Sample size for included RCTs varied from 32 to 2095 with a total of 13 661 participants representing patients from North America, Europe, Asia, and Australia with both solid and hematologic malignant neoplasms (Table). The most frequent treatment was chemotherapy (27 patients [60%]). The meta-analyses and GRADE for available outcomes are in eTable 1 in Supplement 1.

**Table.** Summary of Outcomes for the Addition of Patient-Reported Outcome Measure (PROM) Into Cancer Care Compared With Standard of Care

Outcomes	Anticipated absolute effects (95% CI)		Relative effect (95% CI)	No. of participants (No. of studies)	Certainty of the evidence (GRADE)	Plain language summary
	Risk with standard of care	Risk with the addition of a PROM				
Overall mortality	720 patients per 1000	657 patients per 1000 (600-713)	HR, 0.84 (0.72-0.98)	1289 (3 RCTs)	Moderate (serious reporting bias^a^)	The addition of a PROM was associated with a reduction in overall mortality
HRQoL						
EORTC QLQ-C30 (12 wk follow-up)	NA	MD, 2.45 higher (0.42 higher-4.48 higher)	NR	2113 (6 RCTs)	Moderate (serious inconsistency^b^)	The addition of a PROM was associated with improved HRQoL at 12 wks
EORTC QLQ-C30 (24 wk follow-up)	NA	MD, 1.87 higher (1.21 lower-4.96 higher)	NR	2168 (8 RCTs)	Low (serious risk of bias and serious imprecision^c^^,^^d^)	The addition of a PROM was not associated with HRQoL at 24 wks
EORTC QLQ-C30 (48 wk follow-up)	NA	MD, 0.35 higher (6.31 lower-7.02 higher)	NR	950 (3 RCTs)	Very low (very serious inconsistency and serious imprecision^b^^,^^d^)	The evidence is very uncertain regarding the addition of a PROM on HRQoL at 48 wks
EQ-5D (24 wk follow-up)	NA	MD, 2.58 higher (2.65 lower-7.81 higher)	NR	1135 (3 RCTs)	Very low (serious inconsistency and very serious imprecision^b^^,^^d^)	There was no association between the addition of a PROM and HRQoL, using EQ5D measured at 24 wks
ED visits	45 persons per 1000	33 persons per 1000 (25-45)	OR, 0.74 (0.54-1.02)	2064 (4 RCTs)	Low (serious inconsistency and serious imprecision^b^^,^^d^)	The addition of a PROM was not associated with a reduction in ED visits
Hospital admissions	24 persons per 1000	21 persons per 1000 (17-24)	OR, 0.86 (0.73-1.02)	2954 (5 RCTs)	Low (serious risk of bias and serious imprecision^c^^,^^d^)	The addition of a PROM was not associated with a reduction in hospital admissions

Abbreviations: ED, emergency department; EORTC QLQ-C30, European Organization for Research and Treatment of Cancer, Core Quality of Life questionnaire; EQ-5D, EuroQol 5 Dimension; GRADE, Grading of Recommendations Assessment, Development and Evaluation; HR, hazard ratio; HRQoL, health-related quality of life; MD, mean difference; NA, not applicable; NR, not reported; OR, odds ratio; RCT, randomized clinical trial.

^a^ Potential reporting bias with only 3 of the 45 trials reporting overall mortality.

^b^ Unexplained inconsistency (large heterogeneity, point estimates vary considerably, and confidence intervals have appreciable nonoverlap).

^c^ Serious concerns for risk of bias, due to the selection of the reported result and/or due to bias arising from the randomization process.

^d^ Boundaries of 95% CIs include both important benefit and important harm.

### Survival

**Figure 2.**
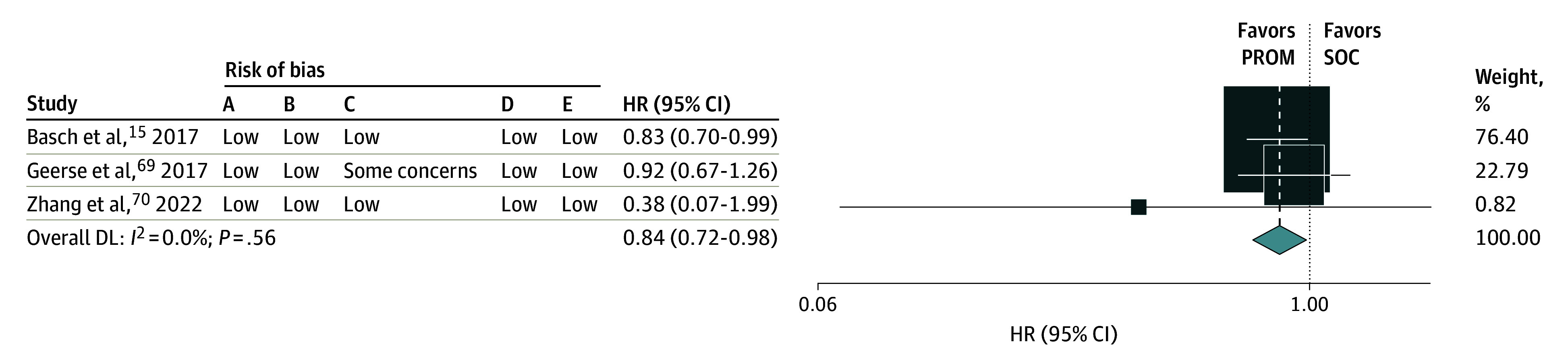
Forest Plot and Risk of Bias for Overall Survival Weights are from random-effects model. Risk of bias categories included: A, random sequence generation; B, allocation concealment; C, masking of participants and personnel; D, incomplete outcome bias; E, selective reporting. DL indicates DerSimonian-Laird random effects meta-analysis; HR, hazard ratio; PROM, patient-reported outcome measures; SOC, standard of care.

Of the 45 RCTs, 4 studies^15,57,69,70^ reported overall mortality; however, only 3 (1289 patients) included data for meta-analyses. The pooled meta-analysis for overall mortality demonstrated that the addition of a PROM likely improves overall mortality (hazard ratio [HR], 0.84; 95% CI, 0.72-0.98; *I^2^* = 0%; *P* for heterogeneity = .55) (moderate certainty) (Figure 2).

### Health-Related Quality of Life

**Figure 3.**
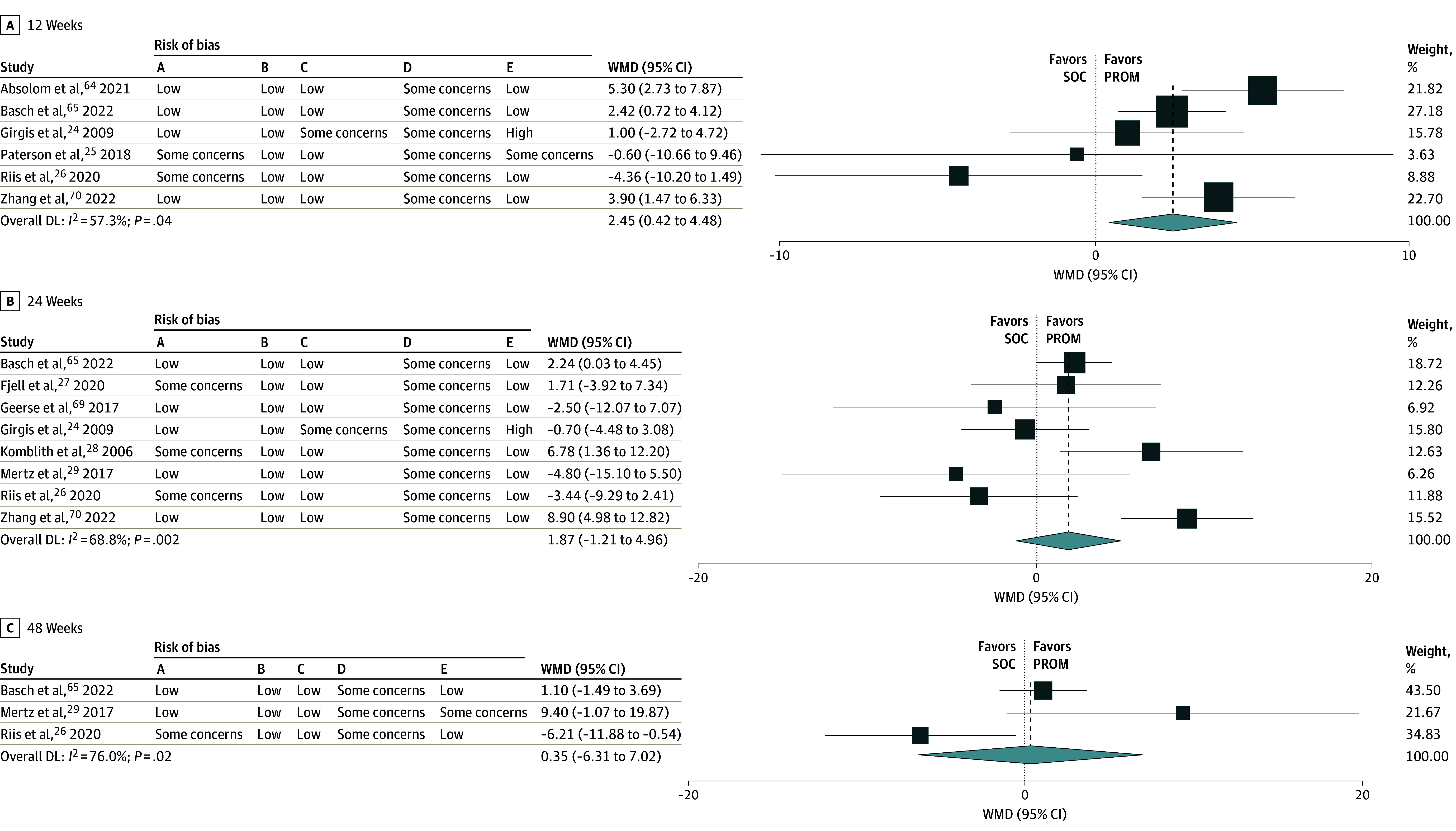
Forest Plots and Risk of Bias for QLQ-C30 Weights are from random-effects model. Risk of bias categories included: A, random sequence generation; B, allocation concealment; C, masking of participants and personnel; D, incomplete outcome bias; E, selective reporting. DL indicates ; PROM, patient-reported outcome measures; QLQ-C30, European Organization for Research and Treatment of Cancer Core Quality of Life questionnaire; SOC, standard of care; WMD, weighted mean difference.

Of the 45 RCTs, 25 studies^16,24,25,26,27,28,29,30,36,37,38,41,50,51,57,58,59,60,62,63,64,65,68,69,70^ reported HRQoL outcomes, using different measures at different time points (eTable 2 in Supplement 1). Six studies^24,25,26,64,65,70^ (2073 participants) measured HRQoL using the European Organization for Research and Treatment of Cancer Core Quality of Life questionnaire (QLQ-C30) at 12 weeks and were included in the pooled meta-analysis. The addition of a PROM was likely to improve HRQoL at 12 weeks (mean difference [MD], 2.45; 95% CI, 0.42-4.48; *I^2^* = 57.3%; *P* for heterogeneity = .04) (moderate certainty) (Figure 3A).

Nine studies^24,25,27,28,30,31,32,33,68^ (1957 participants) measured HRQoL using QLQ-C30 at 24 weeks. One study did not include baseline scores. Eight studies^24,25,27,28,30,31,32,33^ were included in the pooled meta-analysis. Improvements in HRQoL with the addition of a PROM were not significant at 24 weeks (MD, 1.87; 95% CI, −1.21 to 4.96; *I^2^* = 0%; *P* for heterogeneity = .55) (low certainty) (Figure 3B).

Three studies^27,30,33^ (807 participants) measured HRQoL using QLQ-C30 at 48 weeks and were included in the pooled meta-analysis. The evidence was very uncertain about the outcomes associated with the addition of a PROM at 48 weeks (MD, 0.35; 95% CI, −6.31 to 7.02; *I^2^* = 76.0%; *P* for heterogeneity = .02) (very low certainty) (Figure 3C).

Three studies^16,63,69^ (674 participants) measured HRQoL using EuroQol Group 5 Dimension questionnaire (EQ-5D) at 24 weeks and were included in the pooled meta-analysis. The evidence is very uncertain about the outcomes associated with the addition of a PROM using the EQ5D measure (MD, 2.58; 95% CI, −2.65 to 7.81; *I^2^* = 36.5%; *P* for heterogeneity = .21) (very low certainty) (eFigure 1 in Supplement 1).

### Health Care Resource Utilization

**Figure 4.**
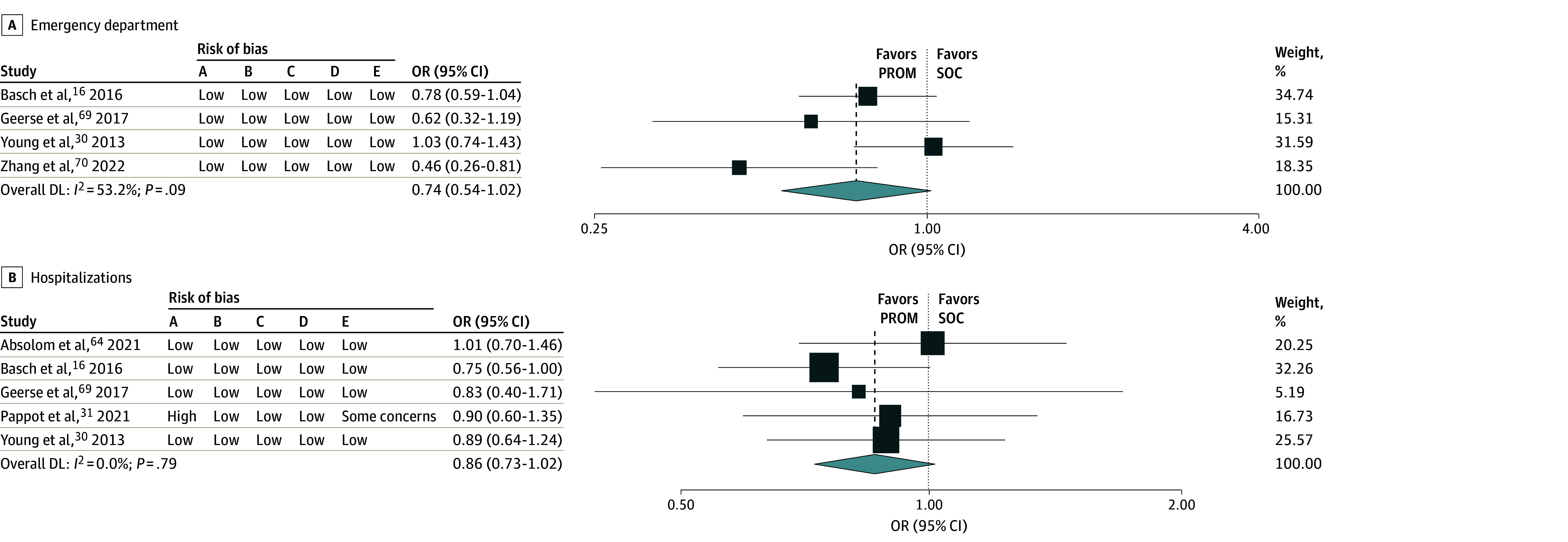
Forest Plots and Risk of Bias for Emergency Department Visits and Hospitalizations Weights are from random-effects model. Risk of bias categories included: A, random sequence generation; B, allocation concealment; C, masking of participants and personnel; D, incomplete outcome bias; E, selective reporting. DL indicates ; OR, odds ratio; PROM, patient-reported outcome measures; SOC, standard of care.

Of the 45 RCTs, 6 studies^16,30,31,64,69,70^ reported ED visits and number of hospitalizations. Four studies^16,30,69,70^ (2064 participants) were included in the pooled ED visits meta-analysis. The addition of a PROM was not associated with a reduction in ED visits (odds ratio [OR], 0.74; 95% CI, 0.54-1.02; *I^2^* = 53.2%; *P* = .09) (low certainty) (Figure 4A).

Five studies^16,30,31,64,69^ (2954 participants) were included in the pooled hospitalization meta-analysis. The addition of a PROM was not associated with a reduction in hospital admissions (OR, 0.86; 95% CI, 0.73-1.02; *I^2^* = 0%; *P* = .79) (low certainty) (Figure 4B).

### Subgroup Analysis

We removed studies with overall high risk of bias (eTable 3 in Supplement 1) and repeated the meta-analysis for those with low risk and high risk of bias. Subgroup analyses based on risk of bias were not applicable for EORTC 48 weeks, EQ-5D 24 weeks, and ED visits. Subgroup analyses for risk of bias did not change overall mortality, HRQoL or health care resource utilization outcomes. No analysis met the threshold (*P* < .10) to apply ICEMAN.

The meta-analysis for studies with low risk included: 2 studies^16,70^ were included in the pooled meta-analysis for overall mortality (HR, 0.82; 95% CI, 0.69 to 0.97; *I^2^* = 0%; *P* = .37) (eFigure 2 in Supplement 1). Five studies^25,26,64,65,70^ were included in the pooled meta-analysis for EORTC-QLQC30 at 12 weeks (HR, 2.86; 95% CI, 0.33 to 4.99; *I^2^* = 62.4%; *P* = .03) (eFigure 3 in Supplement 1). Seven studies^26,27,28,29,65,69^ were included in the pooled meta-analysis for EORTC-QLQC30 at 24 weeks (HR, 2.30; 95% CI, −1.20 to 5.80; *I^2^* = 68.8%; *P* = .002) (eFigure 4 in Supplement 1). Four studies^16,30,64,69^ were included in the pooled hospitalization meta-analysis (HR, 0.86; 95% CI, 0.71 to 1.03; *I^2^* = 0%; *P* = .65) (eFigure 5 in Supplement 1).

## Discussion

In our updated systematic review of 45 RCTs, with a total of 13 661 participants, we were able to conduct a meta-analysis from a proportion of the RCTs for patient-reported outcomes (HRQoL), clinician-reported outcomes (mortality), and health care resource utilization outcomes (ED visits and hospitalizations). We found that the integration of a PROM into cancer care was associated with improved all-cause mortality (HR, 0.84; 95% CI, 0.72-0.98) and HRQoL at 12 weeks (MD, 2.45; 95% CI, 0.42-4.48), but was not associated with HRQoL at 24 weeks (MD, 1.87; 95% CI, −1.21 to 4.96; low certainty). There was no association between the addition of a PROM and HRQoL at 48 weeks. The addition of a PROM was not associated with a reduction in ED visits (OR, 0.74; 95% CI, 0.54-1.02) or hospital admissions (OR, 0.86; 95% CI, 0.73-1.02).

We included many studies but were only able to perform a proper meta-analysis of a limited number of trials because of the heterogeneity of their outcomes. Of the 45 RCTs, only 4 studies measured survival. The improvement in overall mortality with the addition of a PROM is largely influenced by 2 studies.^15,69^ In the Basch study,^16^ patients with cancer receiving active cancer therapy were asked to use an app to report the 12 most common symptoms associated with cancer and its therapy. In the Geerse study,^69^ patients with newly diagnosed lung cancer reported symptom distress using a validated instrument (Distress Thermometer and Problem List). These 2 studies support the concept that using a PROM, specifically on patient-reported symptoms, may assist health care professionals to identify patients’ needs and address issues early thereby preventing poor outcomes. If one is considering implementing PROMs in routine practice, patient-reported symptoms might be a good place to start.

HRQoL, an outcome identified as important to patients, was one of the most common outcomes reported. Of the 45 RCTs, 25 reported HRQoL^16,24,25,26,27,28,29,30,36,37,38,41,50,51,57,58,59,60,62,63,64,65,68,69,70^ as an outcome. However, there was marked variability in the questionnaires used and timing of their administration. Because of the variability, we were only able to conduct meta-analyses on a proportion of HRQoL outcomes. Three studies^64,65,70^ contributed the most to the associations with HRQoL, specifically EORTC measured at 12 weeks. They all used patient-reported symptom monitoring as the intervention,^64,65,70^ again suggesting that asking patients to report their symptoms may lead to an earlier response to symptoms and improvements in HRQoL.

The addition of a PROM may result in a reduction in ED visits and hospital admissions. Only 6 of the 45 RCTs reported ED and hospitalization outcomes.^16,30,31,64,69,70^ There was considerable variability in the timeframe of data collection in these studies, perhaps limiting the certainty of the evidence. In addition to the toxic effects of cancer therapy, there is a burden associated with therapy, requiring multiple scheduled and unscheduled visits to hospital, a burden to patients and their caregivers that has been referred to as time toxicity.^71^ In a health care system with finite resources, hospital resource utilization is also an important outcome for hospital administration.

Multiple studies in this systematic review collected PROMs electronically. This lends itself to the potential for the integration of digital health tools into oncology care. Patient-reported symptoms and other PROMs are an integral component of remote patient monitoring, which can also include vital sign monitoring. Remote patient-monitoring in addition to clinician interactive care, could help anticipate and reduce toxic effects and therapy-related sequalae, improve patient well-being, and potentially reduce hospital resource utilization and treatment burden.

This systematic review and meta-analysis were conducted with rigor using GRADE methodology to assess the certainty of the evidence. In our initial search, we included observational studies in addition to RCTs. Given the large number of available RCTs providing sufficient data for robust meta-analyses, in addition to the advantages of RCTs in terms of internal validity and control over confounding variables, we focused on RCTs only.

This review focused on objectively measured outcomes of integrating PROMS into the clinical care of patients with cancer. When patients systematically report their symptoms and those symptoms are shared with their clinicians, it helps facilitate discussion. In a 2018 review on the use of PROMs, Greenhalgh et al^72^ suggested that in addition to facilitating clinician discussions, the act of completing PROMs prompts the patient to self-reflect on and feel open to discussing their symptoms with a clinician. They also identified that although oncology clinicians are comfortable with managing symptoms, they are not as comfortable with managing issues related to HRQoL or mental health. There is an important role for PROM integration, specifically patient-reported symptoms into oncology care. Studies have demonstrated feasibility in implementing patient-reported symptom reporting in patients on active anticancer therapy,^73,74^ further evidence that PROMs should be adopted into routine oncology care with quality initiatives for standardized implantation and outcome measurements.

### Limitations

Despite the strengths of this study, there are limitations. Similar to the prior review, due to the variability of data collection, measures used, and how results are reported, we were unable to perform a meta-analysis for other common outcomes, such as patient-reported symptoms and patient-reported psychological symptoms. In addition, due to the size of the review, granular data about every study is not reported. A major limitation of the available data is the small number of studies that evaluate the associations of PROM integration with important outcomes, such as survival and hospital resource utilization. Due to the heterogeneity of the PROM interventions used, our study does not provide evidence on the optimal strategy to collect PROs in active oncology care.

## Conclusions

The integration of PROMs into cancer care was associated with overall survival and short-term HRQoL but not reductions in ED visits and hospitalizations. In the 45 RCTs measuring the impact of integrating PROMs into cancer care, there was marked variability in the outcomes selected and the timing of their measurement, limiting our ability to comment on the impact on mental health. There is a role to standardize research methodology utilizing PROMs to ensure consistency, comparability, and reliability in evaluating outcomes.

## Notes

Supplement 1. **eAppendix 1.** Initial Search Strategy **eAppendix 2.** Second Search Strategy **eFigure 1.** Forest Plot and Risk of Bias of EQ5D at 24 Weeks **eTable 1.** Study Characteristics for Included Trials **eTable 2.** Summary of the HRQoL Outcomes With Questionnaire Specific Properties Such as Range of Score and Minimal Important Difference **eTable 3.** Overall Risk of Bias for Each Outcome **eFigure 2.** Forest Plot of Sensitivity Analysis for Overall Survival **eFigure 3.** Forest Plot of Sensitivity Analysis for EORTC-QLQC30 at 12 Weeks **eFigure 4.** Forest Plot of Sensitivity Analysis for EORTC-QLQC30 at 24 Weeks **eFigure 5.** Forest Plot of Sensitivity Analysis for Hospitalizations
eReferences.

Supplement 2.
Data Sharing Statement
