# Supplementary material for: Patient-Reported Outcome Measures in Cancer Care: An Updated Systematic Review and Meta-Analysis
Source: JAMA Netw Open. 2024 Aug 13;7(8):e2424793. doi: 10.1001/jamanetworkopen.2024.24793 (PMC11322847; doi:10.1001/jamanetworkopen.2024.24793)
Supplement: Supplement 1. — eAppendix 1. Initial Search Strategy eAppendix 2. Second Search Strategy eFigure 1. Forest Plot and Risk of Bias of EQ5D at 24 Weeks eTable 1. Study Characteristics for Included Trials eTable 2. Summary of the HRQoL Outcomes With Questionnaire Specific Properties Such as Range of Score and Minimal Important Difference eTable 3. Overall Risk of Bias for Each Outcome eFigure 2. Forest Plot of Sensitivity Analysis for Overall Survival eFigure 3. Forest Plot of Sensitivity Analysis for EORTC-QLQC30 at 12 Weeks eFigure 4. Forest Plot of Sensitivity Analysis for EORTC-QLQC30 at 24 Weeks eFigure 5. Forest Plot of Sensitivity Analysis for Hospitalizations eReferences. [file jamanetwopen-e2424793-s001.pdf]

Supplemental Online Content

Balitsky AK, Rayner D, Britto J, et al. Patient-reported outcome measures in cancer care. *JAMA Netw Open.* 2024;7(8):e2424793. doi:10.1001/jamanetworkopen.2024.24793

eAppendix 1. Initial Search Strategy

eAppendix 2. Second Search Strategy

eFigure 1. Forest Plot and Risk of Bias of EQ5D at 24 Weeks

eTable 1. Study Characteristics for Included Trials

eTable 2. Summary of the HRQoL Outcomes With Questionnaire Specific Properties Such as Range of Score and Minimal Important Difference

eTable 3. Overall Risk of Bias for Each Outcome

eFigure 2. Forest Plot of Sensitivity Analysis for Overall Survival

eFigure 3. Forest Plot of Sensitivity Analysis for EORTC-QLQC30 at 12 Weeks

eFigure 4. Forest Plot of Sensitivity Analysis for EORTC-QLQC30 at 24 Weeks

eFigure 5. Forest Plot of Sensitivity Analysis for Hospitalizations

eReferences.

This supplemental material has been provided by the authors to give readers additional information about their work.

© 2024 Balitsky AK et al. *JAMA Network Open.*

eAppendix 1. Initial Search Strategy

June 24, 2021

MEDLINE	2030
EMBASE	3875
PsycInfo	462
Central	2707
CINAHL	1436
Subtotal	10510
-dupes	-3354
Total	7156

MEDLINE

Database: OVID Medline Epub Ahead of Print, In-Process & Other Non-Indexed Citations, Ovid MEDLINE(R) Daily and Ovid MEDLINE(R) 1946 to Present

Search Strategy:

----

1 exp Neoplasms/ (3489255)

2 (cancer* or neoplasm* or carcinoma* or oncol* or malignan* or tumor* or leukemia* or leukaemia* or sarcoma* or lymphoma* or melanoma* or blastoma* or myeloma*).mp. (4606954)

3 1 or 2 (4799855)

4 (patient reported outcomes or patient reported outcome or patient based outcome or patient reported outcome measure$).mp. (27045)

5 (inventory or instrument* or measure* or self-report*).ti,ab. (3825061)

6 4 and 5 (17872)

7 3 and 6 (3253)

8 Epidemiologic Studies/ (8708)

9 exp Case-Control Studies/ (1189370)

10 exp Cohort Studies/ (2159240)

11 Case control.tw. (134508)

12 (cohort adj (study or studies)).tw. (238711)

13 Cohort analy$.tw. (9174)

14 (Follow up adj (study or studies)).tw. (51361)

15 (observational adj (study or studies)).tw. (123476)

© 2024 Balitsky AK et al. *JAMA Network Open.*

16 Longitudinal.tw. (268228)

17 Retrospective.tw. (597112)

18 Cross sectional.tw. (400545)

19 Cross-sectional studies/ (372470)

20 or/8-19 (3264389)

21 exp animals/ not humans.sh. (4849833)

22 20 not 21 (3196743)

23 7 and 22 (1296)

24 randomized controlled trial.pt. (534665)

25 controlled clinical trial.pt. (94229)

26 randomi?ed.ab. (626711)

27 placebo.ab. (219040)

28 drug therapy.fs. (2336519)

29 randomly.ab. (359969)

30 trial.ab. (556534)

31 groups.ab. (2209788)

32 or/24-31 (5054529)

33 exp animals/ not humans.sh. (4849833)

34 32 not 33 (4396229)

35 7 and 34 (1463)

36 23 or 35 (2191)

37 limit 36 to yr="2012 -Current" (2030)

EMBASE (OVID)

Database: Embase <1974 to 2021 June 23>

Search Strategy:

----

1 exp malignant neoplasm/ (3666710)

© 2024 Balitsky AK et al. *JAMA Network Open.*

2 (cancer* or neoplasm* or carcinoma* or oncol* or malignan* or tumor* or leukemia* or leukaemia* or sarcoma* or lymphoma* or melanoma* or blastoma* or myeloma*).mp. (6022711)

3 1 or 2 (6089519)

4 (patient reported outcomes or patient reported outcome or patient based outcome or patient reported outcome measure$).mp. (49934)

5 (inventory or instrument* or measure* or self-report*).ti,ab. (4948202)

6 4 and 5 (31490)

7 3 and 6 (6768)

8 clinical study/ (155826)

9 case control study/ (174101)

10 family study/ (25360)

11 longitudinal study/ (157330)

12 retrospective study/ (1093385)

13 prospective study/ (693562)

14 randomized controlled trials/ (205892)

15 13 not 14 (685701)

16 cohort analysis/ (720879)

17 (Cohort adj (study or studies)).mp. [mp=title, abstract, heading word, drug trade name, original title, device manufacturer, drug manufacturer, device trade name, keyword, floating subheading word, candidate term word] (355608)

18 (Case control adj (study or studies)).mp. [mp=title, abstract, heading word, drug trade name, original title, device manufacturer, drug manufacturer, device trade name, keyword, floating subheading word, candidate term word] (233699)

19 (follow up adj (study or studies)).mp. [mp=title, abstract, heading word, drug trade name, original title, device manufacturer, drug manufacturer, device trade name, keyword, floating subheading word, candidate term word] (70084)

20 (observational adj (study or studies)).mp. [mp=title, abstract, heading word, drug trade name, original title, device manufacturer, drug manufacturer, device trade name, keyword, floating subheading word, candidate term word] (293105)

21 (epidemiologic$ adj (study or studies)).mp. [mp=title, abstract, heading word, drug trade name, original title, device manufacturer, drug manufacturer, device trade name, keyword, floating subheading word, candidate term word] (116891)

© 2024 Balitsky AK et al. *JAMA Network Open.*

22 (cross sectional adj (study or studies)).mp. [mp=title, abstract, heading word, drug trade name, original title, device manufacturer, drug manufacturer, device trade name, keyword, floating subheading word, candidate term word] (464847)

23 or/8-12,15-22 (3278054)

24 7 and 23 (2301)

25 randomized controlled trial/ (663283)

26 Controlled clinical study/ (464263)

27 random$.ti,ab. (1678708)

28 randomization/ (91086)

29 intermethod comparison/ (273060)

30 placebo.ti,ab. (325879)

31 (compare or compared or comparison).ti. (540864)

32 ((evaluated or evaluate or evaluating or assessed or assess) and (compare or compared or comparing or comparison)).ab. (2324141)

33 (open adj label).ti,ab. (88151)

34 ((double or single or doubly or singly) adj (blind or blinded or blindly)).ti,ab. (245951)

35 double blind procedure/ (185419)

36 parallel group$1.ti,ab. (27710)

37 (crossover or cross over).ti,ab. (111543)

38 ((assign$ or match or matched or allocation) adj5 (alternate or group$1 or intervention$1 or patient$1 or subject$1 or participant$1)).ti,ab. (357548)

39 (assigned or allocated).ti,ab. (421582)

40 (controlled adj7 (study or design or trial)).ti,ab. (382208)

41 (volunteer or volunteers).ti,ab. (258723)

42 human experiment/ (550059)

43 trial.ti. (332237)

44 or/25-43 (5437627)

45 (random$ adj sampl$ adj7 ("cross section$" or questionnaire$1 or survey$ or database$1)).ti,ab. not (comparative study/ or controlled study/ or randomi?ed controlled.ti,ab. or randomly assigned.ti,ab.) (8620)

© 2024 Balitsky AK et al. *JAMA Network Open.*

46 Cross-sectional study/ not (randomized controlled trial/ or controlled clinical study/ or controlled study/ or randomi?ed controlled.ti,ab. or control group$1.ti,ab.) (272969)

47 (((case adj control$) and random$) not randomi?ed controlled).ti,ab. (18630)

48 (Systematic review not (trial or study)).ti. (178809)

49 (nonrandom$ not random$).ti,ab. (17084)

50 "Random field$".ti,ab. (2519)

51 (random cluster adj3 sampl$).ti,ab. (1369)

52 (review.ab. and review.pt.) not trial.ti. (900966)

53 "we searched".ab. and (review.ti. or review.pt.) (37262)

54 "update review".ab. (116)

55 (databases adj4 searched).ab. (43608)

56 (rat or rats or mouse or mice or swine or porcine or murine or sheep or lambs or pigs or piglets or rabbit or rabbits or cat or cats or dog or dogs or cattle or bovine or monkey or monkeys or trout or marmoset$1).ti. and animal experiment/ (1114266)

57 Animal experiment/ not (human experiment/ or human/) (2338374)

58 or/45-57 (3731597)

59 44 not 58 (4828779)

60 7 and 59 (2707)

61 24 or 60 (4136)

62 limit 61 to yr="2012 -Current" (3875)

PsycInfo (OVID)

Database: APA PsycInfo <1806 to June Week 2 2021>

Search Strategy:

----

1 exp neoplasms/ (54786)

2 (cancer* or neoplasm* or carcinoma* or oncol* or malignan* or tumor* or leukemia* or leukaemia* or sarcoma* or lymphoma* or melanoma* or blastoma* or myeloma*).mp. (93452)

3 1 or 2 (93746)

4 patient reported outcome measures/ (389)

5 (patient reported outcomes or patient reported outcome or patient based outcome or patient reported outcome measure$).mp. (3622)

6 (inventory or instrument* or measure* or self-report*).ti,ab. (984915)

7 5 and 6 (2787)

8 4 or 7 (2816)

9 3 and 8 (510)

Cochrane Library CENTRAL (Wiley)

Search Name: 2021-06-23 Amaris PROMS

Date Run: 24/06/2021 20:20:37

Comment:

ID	Search	Hits
#1	MeSH descriptor: [Neoplasms] explode all trees	82113
#2	cancer* or neoplasm* or carcinoma* or oncol* or malignan* or tumor* or leukemia* or leukaemia* or sarcoma* or lymphoma* or melanoma* or blastoma* or myeloma*	253639
#3	#1 or #2	257658
#4	"patient reported outcome"	6600
#5	"patient reported outcomes"	6950
#6	"patient based outcome"	41
#7	"patient reported outcome measure*"	308
#8	#4 or #5 or #6 or #7	10170
#9	#3 and #8 in Trials	2937
#10	#9 with Publication Year from 2012 to 2021, in Trials	2707

CINAHL (EBSCO)

#	Query	Results

S59	S41 OR S58	1,436
S58	S13 AND S57	804
S57	S42 OR S43 OR S44 OR S45 OR S46 OR S47 OR S48 OR S49 OR S50 OR S51 OR S52 OR S53 OR S54 OR S55 OR S56	863,550
S56	AB (CLUSTER W3 RCT)	389
S55	MH (CROSSOVER DESIGN) OR MH (COMPARATIVE STUDIES)	390,888
S54	AB (CONTROL W5 GROUP)	121,670
S53	PT (randomized controlled trial)	129,455
S52	MH (placebos)	12,905
S51	MH (sample size) AND AB (assigned OR allocated OR control)	4,159
S50	TI (trial)	150,765
S49	AB (random*)	340,663
S48	TI (randomised OR randomized)	116,517
S47	(MH "Cluster Sample")	4,771
S46	(MH "Pretest-Posttest Design")	46,444
S45	(MH "Random Assignment")	68,154
S44	(MH "Single-Blind Studies")	14,895
S43	(MH "Double-Blind Studies")	50,589
S42	(MH "Randomized Controlled Trials")	115,887
S41	S13 AND S40	1,043
S40	S14 OR S15 OR S16 OR S17 OR S18 OR S19 OR S20 OR S21 OR S22 OR S23 OR S24 OR S25 OR S26 OR	1,421,217

© 2024 Balitsky AK et al. *JAMA Network Open.*

	S27 OR S28 OR S29 OR S30 OR S31 OR S32 OR S33 OR S34 OR S35 OR S36 OR S37 OR S38 OR S39	
S39	TI “Descriptive stud*” OR AB “Descriptive stud*”	20,522
S38	TI “ecological stud*” OR AB “ecological stud*”	1,221
S37	TI Correlational OR AB Correlational	9,784
S36	TI “Interrupted time series” OR AB “Interrupted time series”	1,990
S35	TI “Controlled before and after” OR AB “Controlled before and after”	387
S34	TI “adverse effect*” OR AB “adverse effect*”	35,941
S33	TI observational OR AB observational	77,429
S32	TI prospective* OR AB prospective*	227,749
S31	TI retrospective* OR AB retrospective*	228,279
S30	TI longitudinal OR AB longitudinal	94,317
S29	TI “follow up” OR AB “follow up”	268,405
S28	TI “cross sectional” OR AB “cross sectional”	164,597
S27	TI cohort* OR AB cohort*	239,577
S26	TI “case series” OR AB “case series”	25,054
S25	TI “case stud*” OR AB “case stud*”	60,552
S24	TI “case referent” OR AB “case referent*”	117
S23	TI "case control" OR AB "case control"	36,932
S22	TI epidemiologic OR AB epidemiologic	17,714
S21	(MH "Descriptive Research")	87,573
S20	(MH "Ecological Research")	1,267

© 2024 Balitsky AK et al. *JAMA Network Open.*

S19	(MH "Correlational Studies")	27,318
S18	(MH "Retrospective Panel Studies")	201
S17	(MH "Prospective Studies+")	471,729
S16	(MH "Cross Sectional Studies")	208,244
S15	(MH "Case Studies")	24,962
S14	(MH "Case Control Studies+")	87,185
S13	S3 AND S12	2,157
S12	S4 OR S11	10,419
S11	S9 AND S10	9,103
S10	TX inventory or instrument* or measure* or self-report*	1,099,448
S9	S5 OR S6 OR S7 OR S8	13,247
S8	TX patient reported outcome measure*	4,482
S7	TX patient based outcome	144
S6	TX patient reported outcomes	13,119
S5	TX patient reported outcome	13,119
S4	(MH "Patient-Reported Outcomes")	3,413
S3	S1 OR S2	950,358
S2	TX cancer* or neoplasm* or carcinoma* or oncol* or malignan* or tumor* or leukemia* or leukaemia* or sarcoma* or lymphoma* or melanoma* or blastoma* or myeloma*	923,592
S1	(MH "Neoplasms+")	580,938

© 2024 Balitsky AK et al. *JAMA Network Open.*

eAppendix 2. Second Search Strategy
Sept 26 2022

database	Total	Update
MEDLINE	2720	641
EMBASE	5232	1506
PsycInfo	605	101
Central	3434	769
CINAHL	1866	537
Subtotal	13857	3554
-dupes		1049
Total		2505

MEDLINE

Database: OVID Medline Epub Ahead of Print, In-Process & Other Non-Indexed Citations, Ovid MEDLINE(R) Daily and Ovid MEDLINE(R) 1946 to Present
Search Strategy:
----
1 exp Neoplasms/ (3739653)
2 (cancer* or neoplasm* or carcinoma* or oncol* or malignan* or tumor* or leukemia* or leukaemia* or sarcoma* or lymphoma* or melanoma* or blastoma* or myeloma*).mp. (4932103)
3 1 or 2 (5133876)
4 (patient reported outcomes or patient reported outcome or patient based outcome or patient reported outcome measure$).mp. (35148)
5 (inventory or instrument* or measure* or self-report*).ti,ab. (4129657)
6 4 and 5 (23296)
7 3 and 6 (4199)
8 Epidemiologic Studies/ (9182)
9 exp Case-Control Studies/ (1355861)
10 exp Cohort Studies/ (2397905)
11 Case control.tw. (146719)
12 (cohort adj (study or studies)).tw. (286109)
13 Cohort analy$.tw. (10766)
14 (Follow up adj (study or studies)).tw. (54449)
15 (observational adj (study or studies)).tw. (146423)
16 Longitudinal.tw. (301544)
17 Retrospective.tw. (687156)
18 Cross sectional.tw. (469179)
19 Cross-sectional studies/ (440988)
20 or/8-19 (3609613)
21 exp animals/ not humans.sh. (5049594)
22 20 not 21 (3536688)
23 7 and 22 (1705)
24 randomized controlled trial.pt. (577665)

25 controlled clinical trial.pt. (95044)
26 randomi?ed.ab. (689248)
27 placebo.ab. (231999)
28 drug therapy.fs. (2532652)
29 randomly.ab. (392069)
30 trial.ab. (617811)
31 groups.ab. (2412574)
32 or/24-31 (5488384)
33 exp animals/ not humans.sh. (5049594)
34 32 not 33 (4784072)
35 7 and 34 (1934)
36 23 or 35 (2881)
37 limit 36 to yr="2012 -Current" (2720)
38 limit 37 to ed=20210624-20220926 (641)

Embase (Ovid)

Database: Embase <1996 to 2022 September 23>
Search Strategy:
----
1 exp malignant neoplasm/ (3418024)
2 (cancer* or neoplasm* or carcinoma* or oncol* or malignan* or tumor* or leukemia* or leukaemia* or sarcoma* or lymphoma* or melanoma* or blastoma* or myeloma*).mp. (5517495)
3 1 or 2 (5566134)
4 (patient reported outcomes or patient reported outcome or patient based outcome or patient reported outcome measure$).mp. (64282)
5 (inventory or instrument* or measure* or self-report*).ti,ab. (4618315)
6 4 and 5 (40053)
7 3 and 6 (8716)
8 clinical study/ (109681)
9 case control study/ (188489)
10 family study/ (22970)
11 longitudinal study/ (171574)
12 retrospective study/ (1285551)
13 prospective study/ (776942)
14 randomized controlled trials/ (234906)
15 13 not 14 (767551)
16 cohort analysis/ (892178)
17 (Cohort adj (study or studies)).mp. [mp=title, abstract, heading word, drug trade name, original title, device manufacturer, drug manufacturer, device trade name, keyword heading word, floating subheading word, candidate term word] (416794)
18 (Case control adj (study or studies)).mp. [mp=title, abstract, heading word, drug trade name, original title, device manufacturer, drug manufacturer, device trade name, keyword heading word, floating subheading word, candidate term word] (243481)

© 2024 Balitsky AK et al. *JAMA Network Open.*

19 (follow up adj (study or studies)).mp. [mp=title, abstract, heading word, drug trade name, original title, device manufacturer, drug manufacturer, device trade name, keyword heading word, floating subheading word, candidate term word] (59990)
20 (observational adj (study or studies)).mp. [mp=title, abstract, heading word, drug trade name, original title, device manufacturer, drug manufacturer, device trade name, keyword heading word, floating subheading word, candidate term word] (351908)
21 (epidemiologic$ adj (study or studies)).mp. [mp=title, abstract, heading word, drug trade name, original title, device manufacturer, drug manufacturer, device trade name, keyword heading word, floating subheading word, candidate term word] (106643)
22 (cross sectional adj (study or studies)).mp. [mp=title, abstract, heading word, drug trade name, original title, device manufacturer, drug manufacturer, device trade name, keyword heading word, floating subheading word, candidate term word] (545584)
23 or/8-12,15-22 (3628028)
24 7 and 23 (3186)
25 randomized controlled trial/ (683889)
26 Controlled clinical study/ (421838)
27 random$.ti,ab. (1701896)
28 randomization/ (86896)
29 intermethod comparison/ (278169)
30 placebo.ti,ab. (298231)
31 (compare or compared or comparison).ti. (457752)
32 ((evaluated or evaluate or evaluating or assessed or assess) and (compare or compared or comparing or comparison)).ab. (2447463)
33 (open adj label).ti,ab. (99161)
34 ((double or single or doubly or singly) adj (blind or blinded or blindly)).ti,ab. (212502)
35 double blind procedure/ (172786)
36 parallel group$1.ti,ab. (27911)
37 (crossover or cross over).ti,ab. (98043)
38 ((assign$ or match or matched or allocation) adj5 (alternate or group$1 or intervention$1 or patient$1 or subject$1 or participant$1)).ti,ab. (356557)
39 (assigned or allocated).ti,ab. (418563)
40 (controlled adj7 (study or design or trial)).ti,ab. (388163)
41 (volunteer or volunteers).ti,ab. (220970)
42 human experiment/ (461689)
43 trial.ti. (339366)
44 or/25-43 (5299364)
45 (random$ adj sampl$ adj7 ("cross section$" or questionnaire$1 or survey$ or database$1)).ti,ab. not (comparative study/ or controlled study/ or randomi?ed controlled.ti,ab. or randomly assigned.ti,ab.) (8222)
46 Cross-sectional study/ not (randomized controlled trial/ or controlled clinical study/ or controlled study/ or randomi?ed controlled.ti,ab. or control group$1.ti,ab.) (316167)
47 (((case adj control$) and random$) not randomi?ed controlled).ti,ab. (19261)
48 (Systematic review not (trial or study)).ti. (222528)
49 (nonrandom$ not random$).ti,ab. (15523)

© 2024 Balitsky AK et al. *JAMA Network Open.*

50 "Random field$".ti,ab. (2714)
51 (random cluster adj3 sampl$).ti,ab. (1418)
52 (review.ab. and review.pt.) not trial.ti. (992191)
53 "we searched".ab. and (review.ti. or review.pt.) (43710)
54 "update review".ab. (114)
55 (databases adj4 searched).ab. (53651)
56 (rat or rats or mouse or mice or swine or porcine or murine or sheep or lambs or pigs or piglets or rabbit or rabbits or cat or cats or dog or dogs or cattle or bovine or monkey or monkeys or trout or marmoset$1).ti. and animal experiment/ (793661)
57 Animal experiment/ not (human experiment/ or human/) (1647985)
58 or/45-57 (3202329)
59 44 not 58 (4664740)
60 7 and 59 (3541)
61 24 or 60 (5493)
62 limit 61 to yr="2012 -Current" (5232)
63 limit 62 to dc=20210623-20220926 (1506)

PsycInfo (OVID)

Database: APA PsycInfo <1806 to September Week 1 2022>
Search Strategy:
----
1 exp Neoplasms/ (58167)
2 (cancer* or neoplasm* or carcinoma* or oncol* or malignan* or tumor* or leukemia* or leukaemia* or sarcoma* or lymphoma* or melanoma* or blastoma* or myeloma*).mp. [mp=title, abstract, heading word, table of contents, key concepts, original title, tests & measures, mesh word] (102191)
3 1 or 2 (102388)
4 patient reported outcome measures/ (584)
5 (patient reported outcomes or patient reported outcome or patient based outcome or patient reported outcome measure$).mp. (4454)
6 (inventory or instrument* or measure* or self-report*).ti,ab. (1041144)
7 5 and 6 (3409)
8 4 or 7 (3462)
9 3 and 8 (605)
10 limit 9 to yr="2021 -Current" (101)

Cochrane Library (Wiley)
Search Name:
Date Run: 26/09/2022 21:58:20
Comment:

ID Search Hits
#1 MeSH descriptor: [Neoplasms] explode all trees 89520

#2	cancer* or neoplasm* or carcinoma* or oncol* or malignan* or tumor* or leukemia* or leukaemia* or sarcoma* or lymphoma* or melanoma* or blastoma* or myeloma*	274894
#3	#1 or #2	279349
#4	"patient reported outcome"	8415
#5	"patient reported outcomes"	8299
#6	"patient based outcome"	42
#7	"patient reported outcome measure*"	371
#8	#4 or #5 or #6 or #7	12436
#9	#3 and #8 in Trials	3665
#10	#9 with Publication Year from 2012 to 2022, in Trials	3434
#11	#10 with Cochrane Library publication date Between Jun 2021 and Sep 2022	769

CINAHL (EBSCO)

				Tuesday, September 27, 2022 5:04:57 PM
**#**	**Query**	**Limiters/Expanders**	**Last Run Via**	**Results**
S60	S59	Limiters - Published Date: 20210101-20221231<br>Search modes - Boolean/Phrase	Interface - EBSCOhost Research Databases<br>Search Screen - Advanced Search<br>Database - CINAHL	537
S59	S41 OR S58	Search modes - Boolean/Phrase	Interface - EBSCOhost Research Databases<br>Search Screen - Advanced Search<br>Database - CINAHL	1,866
S58	S13 AND S57	Search modes - Boolean/Phrase	Interface - EBSCOhost Research Databases<br>Search Screen - Advanced Search<br>Database - CINAHL	1,032
S57	S42 OR S43 OR S44 OR S45 OR S46 OR S47 OR S48 OR S49 OR S50 OR S51 OR S52 OR S53 OR S54 OR S55 OR S56	Search modes - Boolean/Phrase	Interface - EBSCOhost Research Databases<br>Search Screen - Advanced Search<br>Database - CINAHL	975,522
S56	AB (CLUSTER W3 RCT)	Search modes - Boolean/Phrase	Interface - EBSCOhost Research Databases<br>Search Screen - Advanced Search<br>Database - CINAHL	471
S55	MH (CROSSOVER DESIGN) OR MH (COMPARATIVE STUDIES)	Search modes - Boolean/Phrase	Interface - EBSCOhost Research Databases<br>Search Screen - Advanced Search<br>Database - CINAHL	461,151
S54	AB (CONTROL W5 GROUP)	Search modes - Boolean/Phrase	Interface - EBSCOhost Research Databases<br>Search Screen - Advanced Search<br>Database - CINAHL	137,914

© 2024 Balitsky AK et al. *JAMA Network Open.*

S53	PT (randomized controlled trial)	Search modes - Boolean/Phrase	Interface - EBSCOhost Research Databases<br>Search Screen - Advanced Search<br>Database - CINAHL	145,64<br>6
S52	MH (placebos)	Search modes - Boolean/Phrase	Interface - EBSCOhost Research Databases<br>Search Screen - Advanced Search<br>Database - CINAHL	13,445
S51	MH (sample size) AND AB (assigned OR allocated OR control)	Search modes - Boolean/Phrase	Interface - EBSCOhost Research Databases<br>Search Screen - Advanced Search<br>Database - CINAHL	4,379
S50	TI (trial)	Search modes - Boolean/Phrase	Interface - EBSCOhost Research Databases<br>Search Screen - Advanced Search<br>Database - CINAHL	169,85<br>6
S49	AB (random*)	Search modes - Boolean/Phrase	Interface - EBSCOhost Research Databases<br>Search Screen - Advanced Search<br>Database - CINAHL	383,68<br>8
S48	TI (randomised OR randomized)	Search modes - Boolean/Phrase	Interface - EBSCOhost Research Databases<br>Search Screen - Advanced Search<br>Database - CINAHL	131,82<br>2
S47	(MH "Cluster Sample")	Search modes - Boolean/Phrase	Interface - EBSCOhost Research Databases<br>Search Screen - Advanced Search<br>Database - CINAHL	5,074
S46	(MH "Pretest-Posttest Design")	Search modes - Boolean/Phrase	Interface - EBSCOhost Research Databases<br>Search Screen - Advanced Search<br>Database - CINAHL	50,475
S45	(MH "Random Assignment")	Search modes - Boolean/Phrase	Interface - EBSCOhost Research Databases<br>Search Screen - Advanced Search<br>Database - CINAHL	75,602
S44	(MH "Single-Blind Studies")	Search modes - Boolean/Phrase	Interface - EBSCOhost Research Databases<br>Search Screen - Advanced Search<br>Database - CINAHL	15,729
S43	(MH "Double-Blind Studies")	Search modes - Boolean/Phrase	Interface - EBSCOhost Research Databases<br>Search Screen - Advanced Search<br>Database - CINAHL	53,386

© 2024 Balitsky AK et al. *JAMA Network Open.*

S42	(MH "Randomized Controlled Trials")	Search modes - Boolean/Phrase	Interface - EBSCOhost Research Databases Search Screen - Advanced Search Database - CINAHL	132,368
S41	S13 AND S40	Search modes - Boolean/Phrase	Interface - EBSCOhost Research Databases Search Screen - Advanced Search Database - CINAHL	1,372
S40	S14 OR S15 OR S16 OR S17 OR S18 OR S19 OR S20 OR S21 OR S22 OR S23 OR S24 OR S25 OR S26 OR S27 OR S28 OR S29 OR S30 OR S31 OR S32 OR S33 OR S34 OR S35 OR S36 OR S37 OR S38 OR S39	Search modes - Boolean/Phrase	Interface - EBSCOhost Research Databases Search Screen - Advanced Search Database - CINAHL	1,579,404
S39	TI “Descriptive stud*” OR AB “Descriptive stud*”	Search modes - Boolean/Phrase	Interface - EBSCOhost Research Databases Search Screen - Advanced Search Database - CINAHL	22,887
S38	TI “ecological stud*” OR AB “ecological stud*”	Search modes - Boolean/Phrase	Interface - EBSCOhost Research Databases Search Screen - Advanced Search Database - CINAHL	1,439
S37	TI Correlational OR AB Correlational	Search modes - Boolean/Phrase	Interface - EBSCOhost Research Databases Search Screen - Advanced Search Database - CINAHL	10,853
S36	TI “Interrupted time series” OR AB “Interrupted time series”	Search modes - Boolean/Phrase	Interface - EBSCOhost Research Databases Search Screen - Advanced Search Database - CINAHL	2,462
S35	TI “Controlled before and after” OR AB “Controlled before and after”	Search modes - Boolean/Phrase	Interface - EBSCOhost Research Databases Search Screen - Advanced Search Database - CINAHL	415
S34	TI “adverse effect*” OR AB “adverse effect*”	Search modes - Boolean/Phrase	Interface - EBSCOhost Research Databases Search Screen - Advanced Search Database - CINAHL	39,999
S33	TI observational OR AB observational	Search modes - Boolean/Phrase	Interface - EBSCOhost Research Databases Search Screen - Advanced Search Database - CINAHL	90,837

© 2024 Balitsky AK et al. *JAMA Network Open.*

S32	TI prospective* OR AB prospective*	Search modes - Boolean/Phrase	Interface - EBSCOhost Research Databases<br>Search Screen - Advanced Search<br>Database - CINAHL	254,448
S31	TI retrospective* OR AB retrospective*	Search modes - Boolean/Phrase	Interface - EBSCOhost Research Databases<br>Search Screen - Advanced Search<br>Database - CINAHL	267,663
S30	TI longitudinal OR AB longitudinal	Search modes - Boolean/Phrase	Interface - EBSCOhost Research Databases<br>Search Screen - Advanced Search<br>Database - CINAHL	107,828
S29	TI “follow up” OR AB “follow up”	Search modes - Boolean/Phrase	Interface - EBSCOhost Research Databases<br>Search Screen - Advanced Search<br>Database - CINAHL	304,403
S28	TI “cross sectional” OR AB “cross sectional”	Search modes - Boolean/Phrase	Interface - EBSCOhost Research Databases<br>Search Screen - Advanced Search<br>Database - CINAHL	195,075
S27	TI cohort* OR AB cohort*	Search modes - Boolean/Phrase	Interface - EBSCOhost Research Databases<br>Search Screen - Advanced Search<br>Database - CINAHL	281,585
S26	TI “case series” OR AB “case series”	Search modes - Boolean/Phrase	Interface - EBSCOhost Research Databases<br>Search Screen - Advanced Search<br>Database - CINAHL	29,124
S25	TI “case stud*” OR AB “case stud*”	Search modes - Boolean/Phrase	Interface - EBSCOhost Research Databases<br>Search Screen - Advanced Search<br>Database - CINAHL	65,635
S24	TI “case referent” OR AB “case referent*”	Search modes - Boolean/Phrase	Interface - EBSCOhost Research Databases<br>Search Screen - Advanced Search<br>Database - CINAHL	122
S23	TI "case control" OR AB "case control"	Search modes - Boolean/Phrase	Interface - EBSCOhost Research Databases<br>Search Screen - Advanced Search<br>Database - CINAHL	41,029
S22	TI epidemiologic OR AB epidemiologic	Search modes - Boolean/Phrase	Interface - EBSCOhost Research Databases<br>Search Screen - Advanced Search<br>Database - CINAHL	18,975

© 2024 Balitsky AK et al. *JAMA Network Open.*

S21	(MH "Descriptive Research")	Search modes - Boolean/Phrase	Interface - EBSCOhost Research Databases<br>Search Screen - Advanced Search<br>Database - CINAHL	94,177
S20	(MH "Ecological Research")	Search modes - Boolean/Phrase	Interface - EBSCOhost Research Databases<br>Search Screen - Advanced Search<br>Database - CINAHL	1,433
S19	(MH "Correlational Studies")	Search modes - Boolean/Phrase	Interface - EBSCOhost Research Databases<br>Search Screen - Advanced Search<br>Database - CINAHL	29,319
S18	(MH "Retrospective Panel Studies")	Search modes - Boolean/Phrase	Interface - EBSCOhost Research Databases<br>Search Screen - Advanced Search<br>Database - CINAHL	200
S17	(MH "Prospective Studies+")	Search modes - Boolean/Phrase	Interface - EBSCOhost Research Databases<br>Search Screen - Advanced Search<br>Database - CINAHL	510,241
S16	(MH "Cross Sectional Studies")	Search modes - Boolean/Phrase	Interface - EBSCOhost Research Databases<br>Search Screen - Advanced Search<br>Database - CINAHL	237,402
S15	(MH "Case Studies")	Search modes - Boolean/Phrase	Interface - EBSCOhost Research Databases<br>Search Screen - Advanced Search<br>Database - CINAHL	26,139
S14	(MH "Case Control Studies+")	Search modes - Boolean/Phrase	Interface - EBSCOhost Research Databases<br>Search Screen - Advanced Search<br>Database - CINAHL	91,982
S13	S3 AND S12	Search modes - Boolean/Phrase	Interface - EBSCOhost Research Databases<br>Search Screen - Advanced Search<br>Database - CINAHL	2,802
S12	S4 OR S11	Search modes - Boolean/Phrase	Interface - EBSCOhost Research Databases<br>Search Screen - Advanced Search<br>Database - CINAHL	13,647
S11	S9 AND S10	Search modes - Boolean/Phrase	Interface - EBSCOhost Research Databases<br>Search Screen - Advanced Search<br>Database - CINAHL	11,768

© 2024 Balitsky AK et al. *JAMA Network Open.*

S10	TX inventory or instrument* or measure* or self-report*	Search modes - Boolean/Phrase	Interface - EBSCOhost Research Databases<br>Search Screen - Advanced Search<br>Database - CINAHL	1,212,193
S9	S5 OR S6 OR S7 OR S8	Search modes - Boolean/Phrase	Interface - EBSCOhost Research Databases<br>Search Screen - Advanced Search<br>Database - CINAHL	17,545
S8	TX patient reported outcome measure*	Search modes - Boolean/Phrase	Interface - EBSCOhost Research Databases<br>Search Screen - Advanced Search<br>Database - CINAHL	5,960
S7	TX patient based outcome	Search modes - Boolean/Phrase	Interface - EBSCOhost Research Databases<br>Search Screen - Advanced Search<br>Database - CINAHL	149
S6	TX patient reported outcomes	Search modes - Boolean/Phrase	Interface - EBSCOhost Research Databases<br>Search Screen - Advanced Search<br>Database - CINAHL	17,413
S5	TX patient reported outcome	Search modes - Boolean/Phrase	Interface - EBSCOhost Research Databases<br>Search Screen - Advanced Search<br>Database - CINAHL	17,413
S4	(MH "Patient-Reported Outcomes+")	Search modes - Boolean/Phrase	Interface - EBSCOhost Research Databases<br>Search Screen - Advanced Search<br>Database - CINAHL	4,896
S3	S1 OR S2	Search modes - Boolean/Phrase	Interface - EBSCOhost Research Databases<br>Search Screen - Advanced Search<br>Database - CINAHL	1,051,634
S2	TX cancer* or neoplasm* or carcinoma* or oncol* or malignan* or tumor* or leukemia* or leukaemia* or sarcoma* or lymphoma* or melanoma* or blastoma* or myeloma*	Search modes - Boolean/Phrase	Interface - EBSCOhost Research Databases<br>Search Screen - Advanced Search<br>Database - CINAHL	1,022,715
S1	(MH "Neoplasms+")	Search modes - Boolean/Phrase	Interface - EBSCOhost Research Databases<br>Search Screen - Advanced Search<br>Database - CINAHL	636,945

© 2024 Balitsky AK et al. *JAMA Network Open.*

eFigure 1. Forest plot and risk of bias of EQ5D at 24 weeks

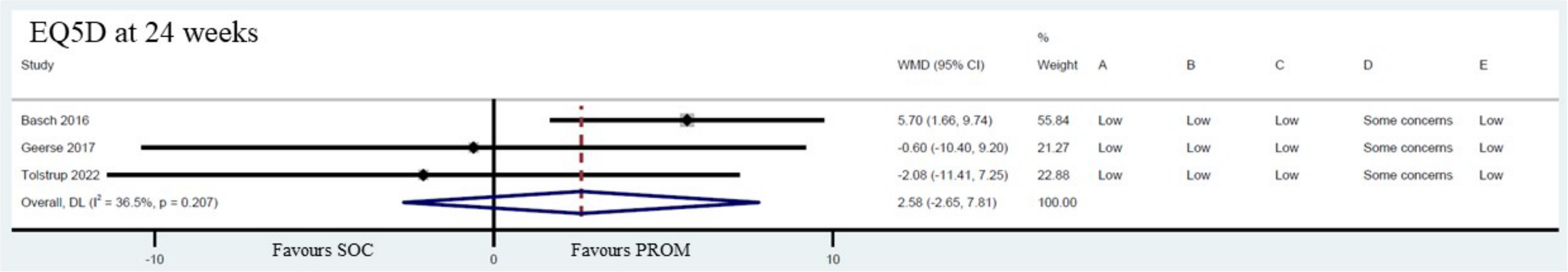

© 2024 Balitsky AK et al. *JAMA Network Open.*

eTable 1. Study characteristics for included trials

Author	Countr(ies)	Timeframe of data collection	Patient population	Type of cancer treatment	No. of patients	PROM intervention	PROM Method	Main findings
Maunsell et al, 1996[1]	Canada	October 1990 and July 1992	breast	chemotherapy, radiation and/or hormone therapy	130 (I); 131 (C)	Monthly telephone screening of distress levels using the GHQ plus psychosocial intervention offered to those with high distress at screening	phone	No difference in HRQoL, physical health, functional status, social and leisure activities, return to work or marital satisfaction
Trowbridge et al, 1997[2]	United States	July to September 1995	mixed cancer diagnosis	any treatment	160 (I) 160 (C)	Pain assessment, describing estimates of pain	paper	Significant difference in clinicians' analgesic prescribing patterns and a decrease in pain in the intervention group
Sarna et al, 1998[3]	United States	NR	lung	chemotherapy	24 (I) 24 (C)	Structured nursing assessments guided by results from SDS	paper	Reduced distress over time in the intervention group
Taenzer et al, 2000[4]	Canada	NR	lung cancer	chemotherapy	27 (I) 26 (C)	Patients completed the EORTC QLQ-C30 and a summary form was available for the clinician	electronic	More quality-of-life issues were addressed in the intervention arm; no difference in patient satisfaction with treatment
McLachlan et al, 2001[5]	Australia	March 1999 - February 2000	Mixed diagnoses	chemotherapy +/- radiotherapy	296 (I) 154 (C)	Multiple measures: CNQ, EORTC QLQ-C30, and BDI; a coordination nurse formulated an individualized management plan based on the issues raised in the summary report of these measures	electronic	No difference between intervention and control arm in cancer needs, quality-of-life, psychosocial functioning or satisfaction with care.
Detmar et al, 2002[6]	Netherlands	June 1996 and June 1998	mixed cancer diagnoses	chemotherapy	114 (I); 100 (C)	Patients completed the EORTC QLQ-C30 and a summary form was available for the clinician	paper	HRQoL issues were discussed more frequently during clinic visits in the intervention arm; no difference between intervention and control arms in HRQoL scores (using SF-36)
Velikova (A) et al, 2004[7]*	United Kingdom	January 2000 and July 2001	mixed cancer diagnosis	any treatment	144 (I); 70 (attention-C); 72 (C)	Patients completed the EORTC QLQ-C30 and HADS and a summary form was available for the clinician.	electronic	Patients in the intervention and attention-control groups demonstrated increased discussions of symptoms and

© 2024 Balitsky AK et al. *JAMA Network Open.*

						There were 2 control groups, the attention-control group completed the questionnaires without feedback to clinicians and the control group did not complete any questionnaire		had better HRQL (using FACT-G) than the control group
Hoekstra et al, 2006[8]	Netherlands	January, 2000 and June, 2002	breast, lung, or GI	palliative chemotherapy	76 (I); 83 (C)	Symptom Monitor: a questionnaire on 10 physicals, symptoms measuring prevalence and severity	paper	Patients in the intervention group had better symptom scores for 9/10 symptoms, 2 were statistically significant (vomiting and constipation)
Kornblith et al, 2006[9]	United States	September 1998 to January 2002	breast, prostate, and colorectal	any treatment	96 (I); 93 (C)	HADS, EORTC QLQ-C30 and a social support survey; with cut-offs to indicate which patients needed a referral to the oncology nurse	phone	Patients in the intervention arm had less anxiety, depression and overall distress
Rosenbloom et al, 2007[10]	United States	1990 - 1992	breast, lung, or colorectal cancer	any treatment	69 (I); 73 (assess-C); 71 (C)	FACT-G and a structured interview about FACT-G responses; assess-C completed FACT-G alone; C received standard of care	paper	Patients in the intervention arm had no improvement in HRQoL or satisfaction
Mills et al, 2009[11]	United Kingdom	April 2005 to January 2007	lung	chemotherapy +/- radiotherapy	57 (I) 58 (C)	Structured HRQoL diary using EORTC QLQ-C30 and LC13	paper	Patients in the intervention group had worse HRQoL; although not statistically significant. There was no difference in satisfaction with care, communication or discussion of patient concerns
Thewes et al, 2009[12]	Australia	NR	mixed cancer diagnosis	surgery, chemotherapy, radiation, or hormone therapy	43 (I) 40 (C)	psychological screen using the DT questionnaire	paper	The intervention arm demonstrated no difference in referrals to psychosocial support staff despite worse DT scores (rural setting cited as limitation to psychosocial support access)
Kearney et al, 2009[13]	United Kingdom	March - September 2006	breast, lung, colorectal cancer	chemotherapy	56 (I); 56 (C)	ASyMS; a remote monitor to manage chemotherapy-related toxicity used patient-reported symptoms (based on CTCAE grading), which prompted	electronic	Participants in the intervention and control arms reported symptoms on paper questionnaires. There was a decrease in fatigue and an increase in hand-foot syndrome in the intervention arm

© 2024 Balitsky AK et al. *JAMA Network Open.*

						escalating alerts to clinicians		
Girgis et al, 2009[14]	Australia	September 2003 and January 2006	breast or colorectal	chemotherapy, radiation, and/or surgery	120 (I-case-worker); 119 (I - clinician); 117 (C)	All groups completed computer-assisted telephone interview, which included HADS and EORTC QLQ-C30 questionnaires. Feedback to clinicians or case-workers were given in two intervention arms	phone	The was increased communication with the healthcare team in the intervention arm; No differences in unmet supportive care needs, HRQoL, anxiety or depression
Ruland et al, 2010[15]	Norway	NR	leukemia or lymphoma	chemotherapy, stem cell transplantation	75 (I) 70 (C)	Interactive tailored patient assessments, provided a summary of patient-reported problems with patient-selected prioritization	electronic	More symptoms addressed in the charts of the intervention group. The intervention arm had a decrease in distress. Over time, there was less need for symptom management in the intervention arm.
Velikova et al, 2010(B)[16]*	United Kingdom	January 2000 and July 2001	mixed cancer diagnosis	any treatment	144 (I); 70 (attention-C); 72 (C)	Patients completed the EORTC QLQ-C30 and HADS and a summary form was available for the clinician.<br>There were 2 control groups, the attention-control group completed the questionnaires without feedback to clinicians and the control group did not complete any questionnaire	electronic	Participants in the intervention arm perceived better continuity of care. No significant effects were found for 'Coordination' or 'Preferences to see usual doctor'.
Berry et al, 2011[17]	United States	April 2009 to June 2011	mixed cancer diagnoses	chemotherapy and/or radiation	327 (I); 333 (C)	self-reported cancer symptoms and quality-of-life using the Electronic Self-Report Assessment–Cancer program	electronic	Patients in the intervention arm demonstrated increased discussions, but there was no difference in length of visits.
Takeuchi et al, 2011[18]*	United Kingdom	January 2000 and July 2001	mixed cancer diagnosis	any treatment	100 (I) 46 (attention-C) 42 (C)	Patients completed the EORTC QLQ-C30 and HADS and a summary form was available for the clinician.<br>There were 2 control groups, the attention-	electronic	Participants in the intervention arm discussed more symptoms over time compared to both control groups

© 2024 Balitsky AK et al. *JAMA Network Open.*

						control group completed the questionnaires without feedback to clinicians and the control group did not complete any questionnaire		
Braeken et al, 2011[19]	Netherlan ds	April 2008 and October 2009	mixed cancer diagnoses	radiotherapy	268 (I); 300 (C) (*in original study*)	Patients completed the Screening Inventory of Psychosocial Problems, a one-page questionnaire to identify psychosocial problems	paper	Feasibility sub-study; participant acceptance rate was 63.6% (21/33)
Cleeland et al, 2011[20]	United States	NR	lung - primary or metastatic	surgery	50 (I); 50 (C)	MDASI, a cancer-related symptom questionnaire, completion with alerts to clinician versus no feedback	electroni c	Participants in the intervention group experienced a reduction in symptoms.
Klinkhammer-Schalke et al, 2012(A)[21]	Germany	September 2004 to August 2006	breast	surgery	99 (I); 100 (C)	Answers to a patient-reported HRQoL questionnaire were analyzed and intervention recommendations were available for the clinician	electroni c	Participants in the intervention arm experienced more improvements in HRQoL compared to control
Nicklasson et al, 2013[22]	Sweden	December 2002 - March 2005	lung or mesotheliom a	chemotherapy +/- radiation	85 (I) 88 (C)	Patients completed the EORTC QLQ-C30 +LC13 questionnaire and their aggregated scores were sent to the clinician	electroni c (control group was paper)	HRQoL measures were similar across groups; Emotional function issues were discussed more frequently in the intervention arm.
Young et al, 2013[23]	Australia	July 2008 - March 2011	colorectal cancer	surgery	387 (I); 369 (C)	The CONNECT intervention is a telephone delivered supportive care intervention administered by centrally located registered nursing	telephon e	There was no difference in ED visits or unplanned hospital admissions, HRQoL score, or distress scores between groups
Snyder et al, 2014[24]	United States	October 2010-October 2012	Breast and prostate	radiation and chemotherapy	74 (I), 77 (I), 73 (I)	Patients completed one of three PROMs (PROMIS, EORTC QLQC30, SCNS-SF34)	electroni c	Participants in the EORTC arm had stronger agreement on the feedback forms compared to the other 2 groups; participants in the SCNS arm reported difficulty in understanding the questions and found the response options confusing

© 2024 Balitsky AK et al. *JAMA Network Open.*

Wheelock et al, 2015[25]	United States	May 2009 - December 2011	breast	any	59 (I); 41 (C)	SIS-NET Web-based system for symptom management, involving -mail invitations to complete the online health questionnaire every 3 months and remote follow-up	electronic	The study was unable to meet its primary outcome of addressing at least 90% of nonurgent symptoms within 3 day. The number of oncology-related clinic visits were similar between groups
Basch et al, 2016(A)[26]	United States	September 2007 - January 2011	breast, genitourinary, gynecologic, or lung	chemotherapy	441 (I); 325 (C)	STAR, a web-based interface of 12 common chemotherapy-associated symptoms adapted from the CTCAE, with email alerts triggered by severity or change in symptoms	electronic	Participants in the intervention group saw more improvements in HRQoL and a reduction in ED visits. Survival benefit reported in separate publication[27]
Mooney et al, 2017[28]	United States	NR	any	chemotherapy	180 (I); 178 (C)	Symptom Care at Home, consisting of 11 chemotherapy-relted symptoms, self-management coaching, automated alerts and a nurse practitioner led remote support system	electronic	Participants in the intervention arm had less symptom severity across all symptoms
Geerse et al, 2017[29]	Netherlands	June 2010 - June 2013	lung	chemotherapy, immunotherapy and/or radiation	110 (I), 113 (C)	Distress thermometer and problem list questionnaire and review with a psychosocial nurse	NR	No difference in HRQoL, anxiety, depression or satisfaction between both groups
Mertz et al, 2017[30]	Denmark	July 2013 - July 2014	breast	chemotherapy and surgery	25 (I); 25 (C)	Screening with EORTC QLQ-C30 and BR23; based on answers from screening, a nurse navigator provided counseling	NR	Participants in the intervention group reported higher satisfaction and lower levels of distress. No differences in HRQoL between groups
Paterson et al, 2018[31]	United Kingdom	March 2016 - April 2017	prostate	androgen deprivation therapy	20 (I); 29 (C)	ThriveCare, a multimodality supportive care intervention, including a holistic needs assessment questionnaire	paper	Participant in the intervention arm had less unmet supportive care needs over time. There was no difference in HRQoL, anxiety, depression, or self-efficacy

© 2024 Balitsky AK et al. *JAMA Network Open.*

Tolstrup et al, 2020[32]**	Denmark	January 2017 - May 2019	Melanoma	immunotherapy	73 (I), 73 (C)	Participants completed a weekly PRO-CTCAE questionnaire on tablets. The software triggered alerts for patients to contact a clinician	electronic	There was no difference in number of grade 3 or 4 adverse events between groups. The number of phone contacts was higher in the intervention arm.
Bryant et al, 2020[33]	United States	May 2015 - June 2017	hematologic	Hematopoietic stem cell transplantation (HCT)	38 (I), 38(C)	Participants admitted to hospital completed a daily PRO-CTCAE questionnaire on tablets. An email with results were sent to a nurse for assessment	electronic	Participants in the intervention arm experienced lower peak symptom burden.
Fjell et al, 2020[34]	Sweden	NR	breast	chemotherapy	75 (I); 75 (C)	Interaktor app, symptom reporting, with alerts sent to clinicians, and education material is sent to patient	electronic	Participants in the intervention arm reposted less symptom prevalence. There was no HRQoL difference between groups, with the exception of a trend to worse emotional function in the intervention arm
Handa et al, 2020[35]	Japan	April 2018 - January 2019	breast	chemotherapy	52 (I); 50 (C)	Breast Cancer Patient Support System application, is an app for symptom reporting based on CTCAE. The app also provides tips on self-care	electronic	There was no difference in HRQoL, anxiety, depression or health literacy between groups
Hentschel et al, 2020[36]	Germany	September 2014 - March 2018	metastatic sarcoma	chemotherapy	38 (I), 41 (C)	PROMs (FACT, HADS, MDASI, BPI) were collected on a tablet, case vignettes created based on results, supportive care recommendations given to patients, graphical representation of result given to clinician	electronic	Participants in the intervention arm had less decline in HRQoL. Mean overall survival was longer in intervention arm, but not significant
Riis et al, 2020[37]***	Denmark	April 2016 - June 2017	breast	endocrine therapy	65 (I), 69 (C)	PROMs as a screening and dialogue tool	electronic	No differences in satisfaction, unmet needs, adherence to treatment or HRQoL
Moore et al, 2020[38]	Australia	May 2017 - October 2018	multiple myeloma	any	16 (I), 16 (C)	Myeloma patient outcome scale, an adaptation of the palliative care outcome scale	electronic or paper	Participants in the intervention arm reported enhanced patient/clinician communication

© 2024 Balitsky AK et al. *JAMA Network Open.*

Rogers et al, 2020[39]	United Kingdom	April 2017 - January 2018	Head and neck	any curative intent	140 (I); 148 (C)	Patient concerns inventory	electronic or paper	Consultations times were similar between groups
Klinkhammer-Schalke et al, 2020 (B)[40]	Germany	January 2014 – October 2015	colorectal	surgery	110 (I); 110 (C)	Answers to a patient-reported HRQoL questionnaire were analyzed and intervention recommendations were available for the clinician	electronic	Over time, participants in the intervention arm had less need for HRQoL-directed therapy
Rodin et al, 2020[41]	Canada	March 2015 - November 2016	Leukemia	chemotherapy	22 (I); 20 (C)	Emotion And Symptom-focused Engagement, a program which includes a weekly physical symptom trigger	paper	Participants in the intervention arm experienced less traumatic stress symptoms and less pain intensity
Absolom et al, 2021[42]	United Kingdom	January 2015 - June 2018	colorectal, breast, or gynecologic	chemotherapy	256 (I); 252 (C)	eRAPID, a patient self-report symptom system	electronic	Participants in the intervention arm had initial improvements physical well-being. There was no difference in hospital admissions
Judge et al, 2021[43]	United States	NR	mixed cancer diagnoses	chemotherapy or radiation	68 (I); 62 (C)	PAIN*Report*it, an extension of the McGill Pain Questionnaire	electronic	Participants in the intervention arm had less current pain, but no difference in worst pain intensity or pain quality
Pappot et al, 2021[44]	Denmark	NR	breast	chemotherapy	347 (I), 335 (C)	PRO-CTCAE completed prior to each cycle of chemotherapy	electronic	There was no difference in treatment adjustments between groups
Riis et al, 2021 (B)[45]***		April 2016 - June 2017	breast	endocrine therapy	65 (I), 69 (C)	PROMs as a screening and dialogue tool	electronic	Participants in the intervention arm had less urgent appointments
Warsame et al, 2022[46]	United States	July 2016- April 2018	multiple myeloma, AL amyloidosis, head and neck cancer and gynecologic malignancies	NR	153 (I); 80 (C)	PRO-QOL system that asked patients what as their biggest concern. It was completed prior to each clinic visit	electronic	There was no difference in quality of life between groups
Basch(B) et al, 2022[47]	United States	October 2017 - March 2020	metastatic cancer	chemotherapy, immunotherapy or targeted therapy	597 (I); 600 (C)	PRO measures including, PRO-CTCAE, oral intake, performance status, falls, and financial challenges,	electronic	Participants in the intervention arm reported improvements in HRQoL, physical function and symptoms control

© 2024 Balitsky AK et al. *JAMA Network Open.*

						completed weekly, with symptom triggers		
Zhang et al, 2022[48]	China	September 2019 - March 2021	mixed cancer diagnoses	immunotherapy +/- targeted or chemotherapy	150 (I); 150 (C)	ePRO app (CTCAE symptoms)	electroni c	Participants in the intervention arm had less serious adverse events, reduced ED visits, better HRQoL. There was no difference in mortality.
Tolstrup et al, 2022[49]**	Denmark	Jan. 2017 - May 2019	Melanoma	Immunotherapy	73 (I); 73 (C)	Participants completed a weekly PRO-CTCAE questionnaire on tablets. The software triggered alerts for patients to contact a clinician	electroni c	Participants in the intervention group saw an improvement in HRQoL as measured by EQ-5D-5, but not FACT-M

I, intervention arm; C, control arm; GHQ, general health questionnaire; HRQoL, health-related quality of life; SDS, Symptom Distress Scale; EORTC QLQ-C30**, European Organization for Research and Treatment of Cancer Quality of Life Questionnaire;** CNQ, Cancer Needs Questionnaire–short form; BDI, Beck depression inventory; SF-36 Medical Outcomes Study 36-item Short Form Health Survey; FACT-G, functional assessment of cancer therapy – general; HADS, hospital anxiety and depression scale; LC13, lung cancer subscale of EORTC QLQ-C30; DT, distress thermometer; ASyMS, advanced symptom management system; CTCAE, common toxicity criteria adverse events; MDASI, M. D. Anderson Symptom Inventory; ED, emergency department; PROMIS, Patient-Reported Outcomes Measurement Information System; SCNS-SF34, Supportive Care Needs Survey-Short Form; SIS-NET, System for Individualized Survivorship Care, based on patient self-reported data, with review by Nurse practitioners, targeted Education, and Triage; STAR, Symptom Tracking and Reporting; EQ-5D-5, generic EuroQol EQ-5D Index; BPI, brief pain inventory; eRAPID Electronic patient self-Reporting of Adverse events: Patient Information and advice; PRO-CTCAE, patient-reported outcome common toxicity criteria adverse events; PRO-QOL, patient-reported outcome quality of life

*, **, *** same data set

© 2024 Balitsky AK et al. *JAMA Network Open.*

eTable 2. Summary of the HRQoL outcomes with questionnaire specific properties such as range of score and minimal important difference

Study	HRQoL measure	Range	MID	Units Reported		Baseline	4 weeks	6 weeks	3 months	4 months	6 months	9 months	1 year
**Absolom et al, 2021[42]**	EORTC QLQ-C30	0-100	5-10[50]*	Mean (SD)	SC	79.9 (15)		75.3 (16.8)	71.7 (16.7)	72.1 (17.9)			
					IC	79.2 (15.6)		77.7 (13)	76.3 (13.3)	76 (15.4)			
	EQ5D	0-100	6-8[51]	Mean (SD)	SC	75.2 (18.6)		71.4 (19.5)	68.9 (19.8)	68.7 (20.4)			
					IC	76.3 (18.1)		74 (17.3)	74 (16.6)	75.6 (18)			
	FACT-G	0-108	3-7[52]	Mean (SD)	SC	81.9 (14.1		76.6 (15.7)	74.3 (16.1)	75.7 (16.6)			
					IC	82.9 (14.1)		80 (15.6)	79.2 (15)	78.8 (16.2)			
**Basch et al, 2016[26]**	EQ5D	0-100	6-8[51]	Mean (SD)	SC	86.6 (84.7 to 88.5)					79.5		
					IC	86.2 (84.7 to 87.7)					84.8		
**Basch et al, 2022[47]**	EORTC QLQ-C30	0-100	5-10[50]	Mean (SD)	SC	78.11	80.52		80.03		79.48		77.47
					IC	77	77.18		76.5		76.13		75.26
**Detmar et al, 2002[6]**	COOP	NR	NR	*Completed at different timepoints*									
**Fjell et al, 2020[34]**	EORTC QLQ-C30	0-100	5-10[50]	Mean (SD)	SC	65.65 (24.36)					54.82		
					IC	67.33 (23.76)					58.21		
**Geerse et al, 2017[29]**	EORTC QLQ-C30	0-100	5-10[50]	Mean change (SE)	SC						5.8 (3.6)		
					IC						3.3 (3.3)		
	EQ5D	0-1	0.6-0.8[51]	Mean change (SE)	SC						(-)0.004 (0.03)		
					IC						(-)0.01 (0.04)		
**Girgis et al, 2009[14]**	EORTC QLQ-C30	0-100	5-10[50]	Mean (SD)	SC	73.2 (19.6)			78.3(19.4)		79.2 (79)		
					IC	71.9 (17.6)			76 (16.3)		78.6 (16.7)		
**Hentchel et al, 2020[36]**	FACT-G	0-108	3-7[52]	Mean change	SC	NR						(-) 3.9 (-11.3-3.5)	
					IC	NR						(-)2.4 (-9.2-4.5)	
**Klinkhammer-Schalke et al, 2020[40]**	EORTC QLQ-C30	0-100	5-10[50]	*Reported as less than 50 or above 50*	SC	x			x		x		x and18 m

© 2024 Balitsky AK et al. *JAMA Network Open.*

					IC	x			x		x		x and18 m
**Kornblith et al, 2006[9]**	EORTC QLQ-C30	0-100	5-10[50]	Mean (SD)	SC	61.45 (21.18)					64.79 (20.71)		
					IC	68.99 (18.17)					65.55 (20.40)		
**McLachlan et al, 2001[5]**	EORTC QLQ-C30	0-100	5-10[50]	*mean change reported for domains*	SC	NR		x			x		
					IC			x			x		
						NR							
**Mertz et al, 2017[30]**	EORTC QLQ-C30	0-100	5-10[50]	Mean (SD)	SC	58.0 (26.1)					66.7 (16.2)		67.5 (20.9)
					IC	58.6 (19.2)					62.5 (22.0)		77.5 (19.7)
**Mills et al, 2009[11]**	FACT-LCS	0-144	2-3[53]	Mean change (SD)	SC	NR				(-)6.6 (12.5)			
					IC	NR				0.2 (15.7)			
**Moore et al, 2020[38]**	MyPOS HRQoL	0-135	NA	*Reported in figure*		x					x	x	
						x					x	x	
**Nicklasson et al, 2013[22]**	EORTC QLQ-C30	0-100	5-10[50]	*Completed at different time points*									
**Paterson et al, 2018[31]**	EORTC QLQ-C30	0-100	5-10[50]	Mean (SD)	SC	73.5 (22.2)			79.6 (23.9)				
					IC	76.4 (23.4)			81.9 (19.6)				
**Riis et al, 2020[37]**	EORTC QLQ-C30	0-100	5-10[50]	*Mean scores extracted from a figure*	SC	x			x		x	x	x
					IC	x			x		x	x	x every 3m until 24m
**Rodin et al, 2020[41]**	FACT-Sp	0-48	NR	Mean (SE)	SC	106.71 (6.80)	103.57 (7.71)	103.70 (7.00)					100.61 (7.50)
					IC	103.92 (6.45)	110.14 (6.83)	110.41 (7.29)					114.37 (6.97)
**Rogers et al, 2020[39]**	MyPOS HRQOL	0-135	NR	NA	SC	x	x				x	x (10m)	
					IC								
**Snyder et al, 2014[24]**	EORTC QLQ-C30	0-100	5-10[50]	*Completed at different time points*	SC	NR							

© 2024 Balitsky AK et al. *JAMA Network Open.*

					IC	NR					
**Tolstrup et al, 2022[49]**	EQ5D	0-100	6-8[51]	Mean	SC	85	85	80	0.85		
					IC	85	87	87	0.87		
	FACT-M	0-172	*Reported for subscales[54]*	Mean	SC	142					147
					IC	140					140
**Velikova et al, 2004[7]**	FACT -G	0-108	3-7[52]	Mean (SD)	SC	70.6 (16.59)					
					IC	72.3 (18.56)					
					IC	73.2 (16.64					
**Warsame et al, 2022[46]**	LASA[55]	0-10	NR		SC	x					x
					IC						
**Young et al, 2013[23]**	FACT-C	0-136	5-8[56]	Mean (95% CI)	SC	102.46 (100.11-104.81)	100.4 (98.48-102.31)		103.26 (101.31-105.21)	105.1 (103.14 – 107.07)	
					IC	101.26 (99.1-103.41)	100.61 (98.73-102.49)		103.48 (101.53-105.44)	105.35 (103.3 – 107.4)	
**Zhang et al, 2022[48]**	EORTC QLQ-C30	0-100	5-10[50]*	Mean (SD)	SC	61.3 (12.7)			66.2 (10.2)	74.2 (15.1)	
					IC	60.7 (15.8)			61.7 (12.1)	64.7 (28.5)	

*Across 9 different cancer types

SC, standard care; IC, intervention care; COOP, Dartmouth Primary Care Cooperative information functional health assessment; EORTC QLQ-C30, European Organization for Research and Treatment of Cancer, Core Quality of Life questionnaire; EQ5D: EuroQol- 5 Dimension; FACT-G, Functional Assessment of Cancer Therapy-General; FACT-C, FACT-Colorectal; FACT-LCS, FACT-Lung cancer; FACT-M, FACT-melanoma, FACT-Sp, FACT- Therapy-Spiritual Well-Being Scale; LASA, Linear Analog Self-Assessment; MID, minimal important difference; MyPOS, Myeloma Patient Outcome Scale

© 2024 Balitsky AK et al. *JAMA Network Open.*

eTable 3. Overall risk of bias for each outcome

Study	Overall mortality	EORTC-QLQ C30 at 12 weeks	EORTC-QLQ C30 at 24 weeks	EORTC-QLQ C30 at 48 weeks	EQ5D at 24 weeks	ED visits	Hospital admissions
Kornblith et al, 2006[9]	-	-	High risk of bias	-	-	-	-
Girgis et al, 2009[14]	-	High risk of bias	High risk of bias	-	-	-	-
Young et al, 2013[23]	-	-	-	-	-	Low risk of bias	Low risk of bias
Basch et al, 2017[27]	Low risk of bias	Some concerns	Some concerns	Some concerns	Some concerns	Low risk of bias	Low risk of bias
Geerse et al, 2017[29]	Some concerns	-	Some concerns	-	Some concerns	Low risk of bias	Low risk of bias
Mertz et al, 2017[30]	-	-	Some concerns	High risk of bias	-	-	-
Paterson et al, 2018[31]	-	High risk of bias	-	-	-	-	-
Fjell et al, 2020[34]	-	-	High risk of bias	-	-	-	-
Riis et al, 2020[37]	-	High risk of bias	High risk of bias	High risk of bias	-	-	-
Absolom et al, 2021[42]	-	Some concerns	-	-	-	-	Low risk of bias
Pappot et al, 2021[44]	-	-	-	-	-	-	High risk of bias
Tolstrup et al, 2022[49]	-	-	-	-	Some concerns	-	-
Zhang et al, 2022[48]	Low risk of bias	Some concerns	Some concerns	-	-	Low risk of bias	-

EORTC QLQ-C30: European Organization for Research and Treatment of Cancer, Core Quality of Life questionnaire; EQ5D: EuroQol- 5 Dimension; ED: emergency department

© 2024 Balitsky AK et al. *JAMA Network Open.*

eFigure 2. Forest plot of sensitivity analysis for overall survival

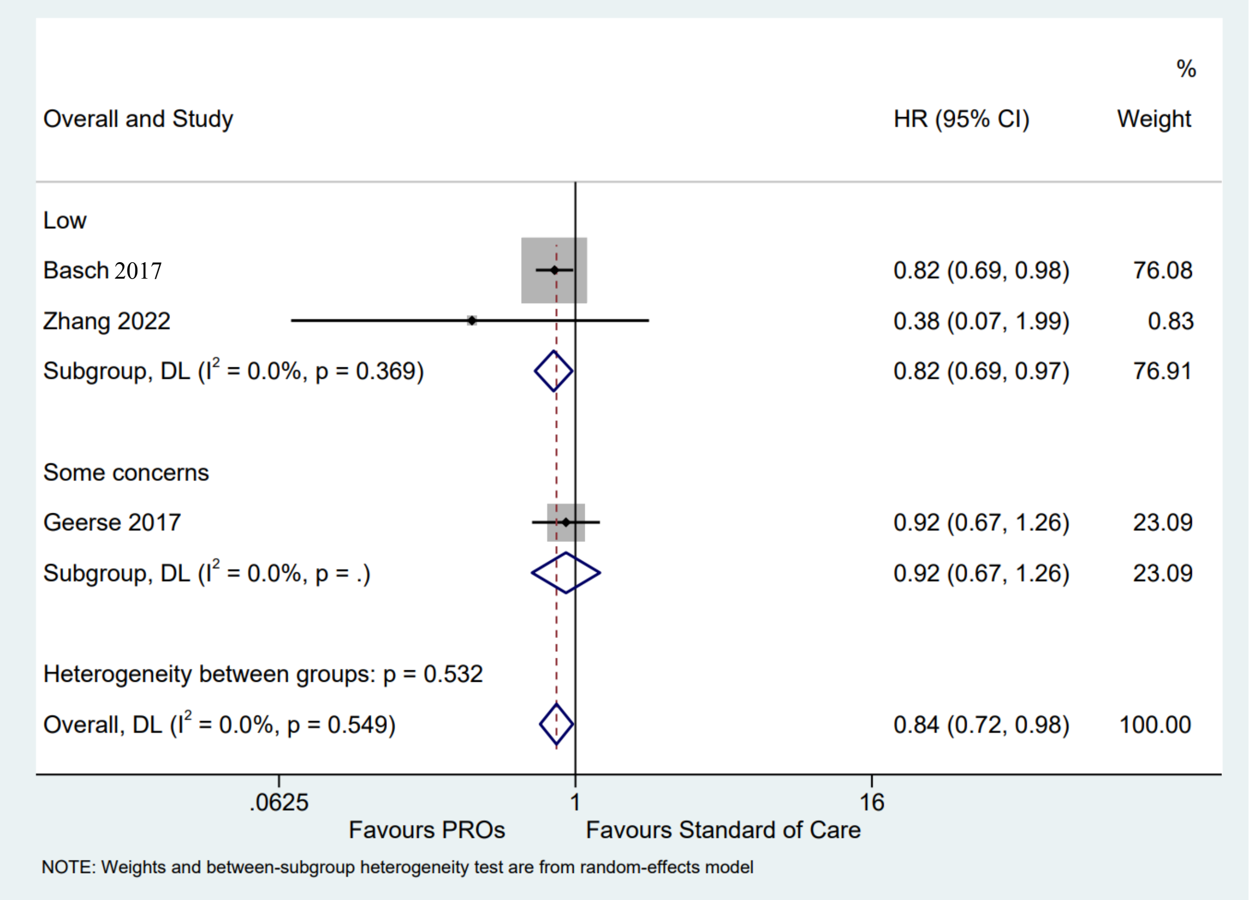

© 2024 Balitsky AK et al. *JAMA Network Open.*

eFigure 3. Forest plot of sensitivity analysis for EORTC-QLQC30 at 12 weeks

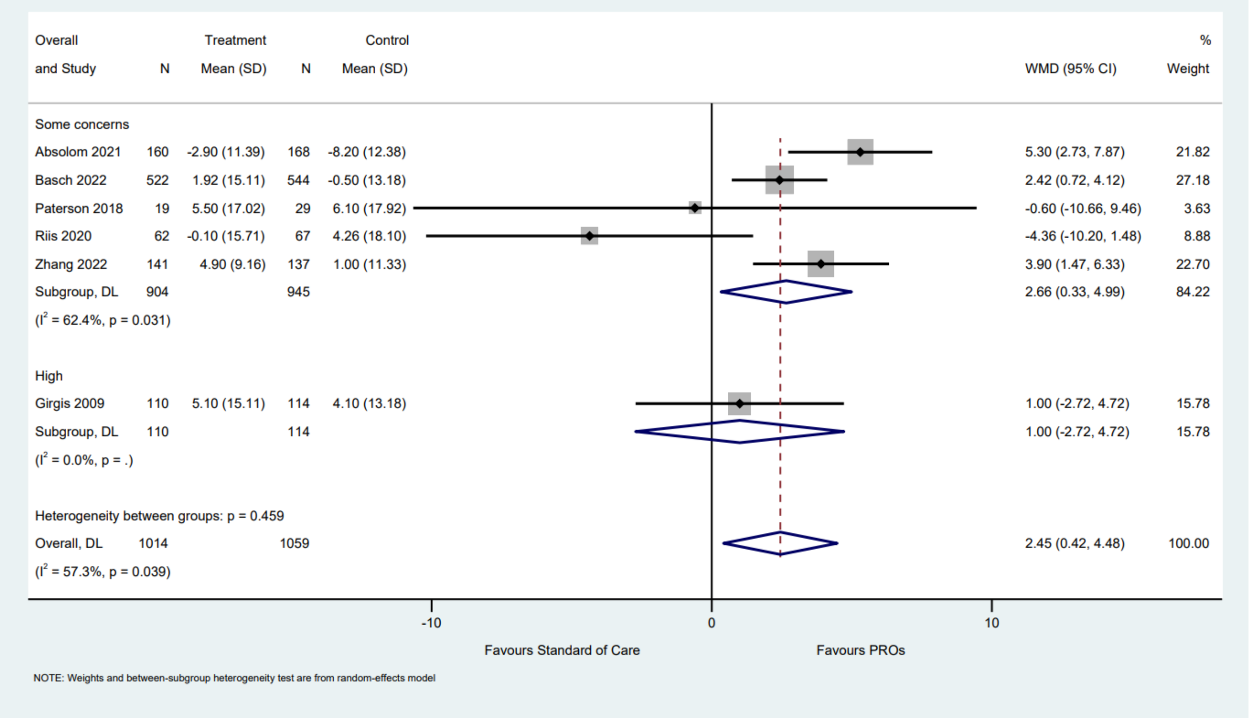

© 2024 Balitsky AK et al. *JAMA Network Open.*

eFigure 4. Forest plot of sensitivity analysis for EORTC-QLQC30 at 24 weeks

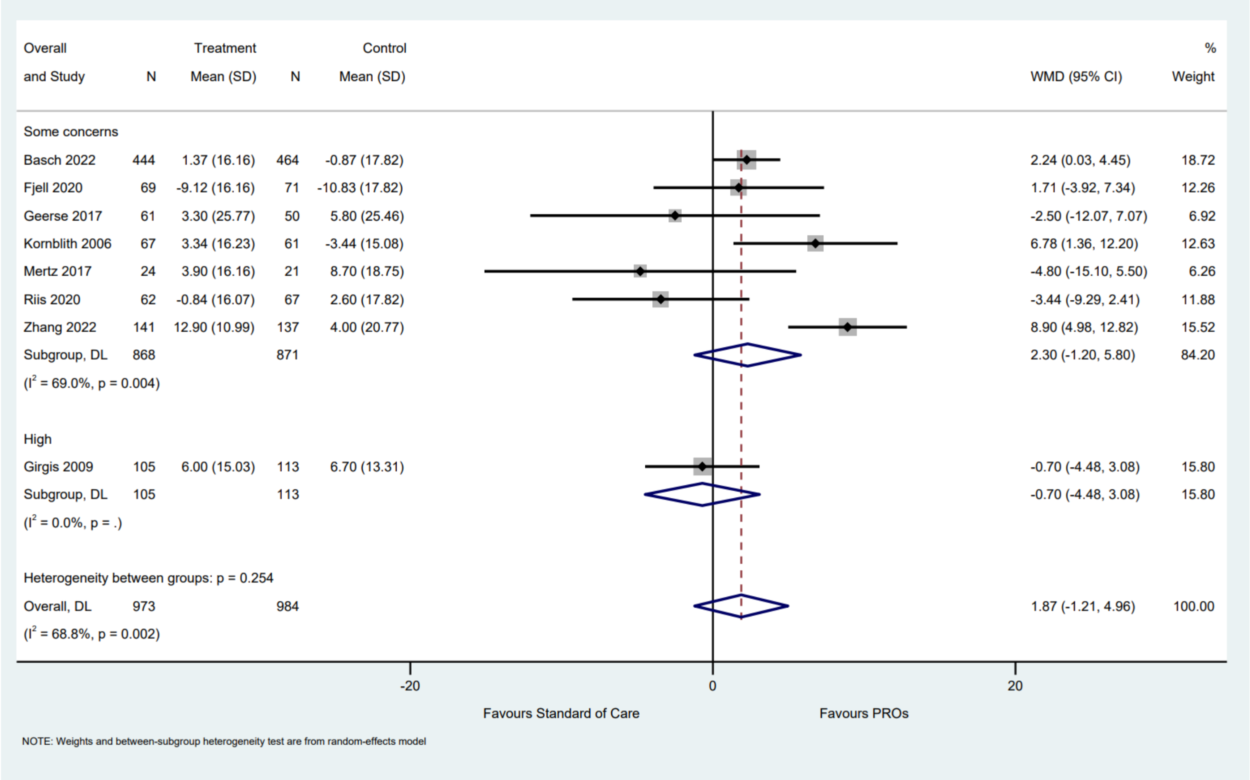

© 2024 Balitsky AK et al. *JAMA Network Open.*

eFigure 5. Forest plot of sensitivity analysis for hospitalizations

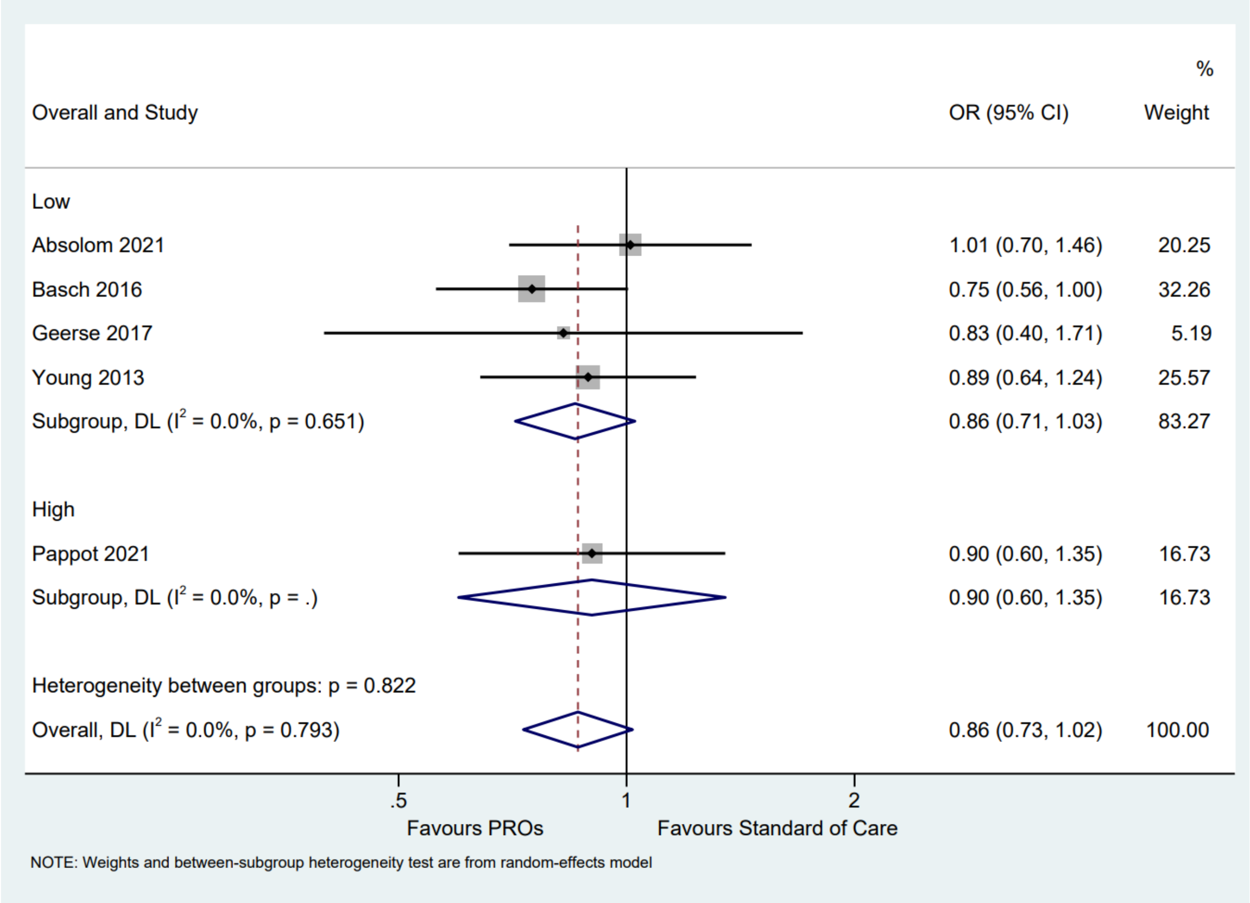

© 2024 Balitsky AK et al. *JAMA Network Open.*

eReferences

1. Maunsell E, Brisson J, Deschênes L, Frasure-Smith N. Randomized trial of a psychologic distress screening program after breast cancer: effects on quality of life. *J Clin Oncol*. Oct 1996;14(10):2747-55. doi:10.1200/jco.1996.14.10.2747
2. Trowbridge R, Dugan W, Jay SJ, et al. Determining the effectiveness of a clinical-practice intervention in improving the control of pain in outpatients with cancer. *Acad Med*. Sep 1997;72(9):798-800. doi:10.1097/00001888-199709000-00016
3. Sarna L. Effectiveness of structured nursing assessment of symptom distress in advanced lung cancer. *Oncol Nurs Forum*. Jul 1998;25(6):1041-8.
4. Taenzer P, Bultz BD, Carlson LE, et al. Impact of computerized quality of life screening on physician behaviour and patient satisfaction in lung cancer outpatients. *Psychooncology*. May-Jun 2000;9(3):203-13. doi:10.1002/1099-1611(200005/06)9:3<203::aid-pon453>3.0.co;2-y
5. McLachlan S-A, Allenby A, Matthews J, et al. Randomized Trial of Coordinated Psychosocial Interventions Based on Patient Self-Assessments Versus Standard Care to Improve the Psychosocial Functioning of Patients With Cancer. *Journal of Clinical Oncology*. 2001;19(21):4117-4125. doi:10.1200/jco.2001.19.21.4117
6. Detmar SB, Muller MJ, Schornagel JH, Wever LD, Aaronson NK. Health-related quality-of-life assessments and patient-physician communication: a randomized controlled trial. *Jama*. Dec 18 2002;288(23):3027-34. doi:10.1001/jama.288.23.3027
7. Velikova G, Booth L, Smith AB, et al. Measuring quality of life in routine oncology practice improves communication and patient well-being: a randomized controlled trial. *J Clin Oncol*. Feb 15 2004;22(4):714-24. doi:10.1200/jco.2004.06.078
8. Hoekstra J, de Vos R, van Duijn NP, Schadé E, Bindels PJ. Using the symptom monitor in a randomized controlled trial: the effect on symptom prevalence and severity. *J Pain Symptom Manage*. Jan 2006;31(1):22-30. doi:10.1016/j.jpainsymman.2005.06.014
9. Kornblith AB, Dowell JM, Herndon JE, 2nd, et al. Telephone monitoring of distress in patients aged 65 years or older with advanced stage cancer: a cancer and leukemia group B study. *Cancer*. Dec 1 2006;107(11):2706-14. doi:10.1002/cncr.22296
10. Rosenbloom SK, Victorson DE, Hahn EA, Peterman AH, Cella D. Assessment is not enough: a randomized controlled trial of the effects of HRQL assessment on quality of life and satisfaction in oncology clinical practice. *Psychooncology*. Dec 2007;16(12):1069-79. doi:10.1002/pon.1184
11. Mills ME, Murray LJ, Johnston BT, Cardwell C, Donnelly M. Does a patient-held quality-of-life diary benefit patients with inoperable lung cancer? *J Clin Oncol*. Jan 1 2009;27(1):70-7. doi:10.1200/jco.2008.17.5687
12. Thewes B, Butow P, Stuart-Harris R. Does routine psychological screening of newly diagnosed rural cancer patients lead to better patient outcomes? Results of a pilot study. *Aust J Rural Health*. Dec 2009;17(6):298-304. doi:10.1111/j.1440-1584.2009.01087.x
13. Kearney N, McCann L, Norrie J, et al. Evaluation of a mobile phone-based, advanced symptom management system (ASyMS) in the management of chemotherapy-related toxicity. *Support Care Cancer*. Apr 2009;17(4):437-44. doi:10.1007/s00520-008-0515-0
14. Girgis A, Breen S, Stacey F, Lecathelinais C. Impact of two supportive care interventions on anxiety, depression, quality of life, and unmet needs in patients with nonlocalized breast and colorectal cancers. *J Clin Oncol*. Dec 20 2009;27(36):6180-90. doi:10.1200/jco.2009.22.8718
15. Ruland CM, Holte HH, Røislien J, et al. Effects of a computer-supported interactive tailored patient assessment tool on patient care, symptom distress, and patients' need for symptom

© 2024 Balitsky AK et al. *JAMA Network Open.*

management support: a randomized clinical trial. *J Am Med Inform Assoc*. Jul-Aug 2010;17(4):403-10. doi:10.1136/jamia.2010.005660
16. Velikova G, Keding A, Harley C, et al. Patients report improvements in continuity of care when quality of life assessments are used routinely in oncology practice: secondary outcomes of a randomised controlled trial. *Eur J Cancer*. Sep 2010;46(13):2381-8. doi:10.1016/j.ejca.2010.04.030
17. Berry DL, Blumenstein BA, Halpenny B, et al. Enhancing patient-provider communication with the electronic self-report assessment for cancer: a randomized trial. *J Clin Oncol*. Mar 10 2011;29(8):1029-35. doi:10.1200/jco.2010.30.3909
18. Takeuchi EE, Keding A, Awad N, et al. Impact of patient-reported outcomes in oncology: a longitudinal analysis of patient-physician communication. *J Clin Oncol*. Jul 20 2011;29(21):2910-7. doi:10.1200/jco.2010.32.2453
19. Braeken AP, Kempen GI, Eekers D, van Gils FC, Houben RM, Lechner L. The usefulness and feasibility of a screening instrument to identify psychosocial problems in patients receiving curative radiotherapy: a process evaluation. *BMC Cancer*. Nov 8 2011;11:479. doi:10.1186/1471-2407-11-479
20. Cleeland CS, Wang XS, Shi Q, et al. Automated symptom alerts reduce postoperative symptom severity after cancer surgery: a randomized controlled clinical trial. *J Clin Oncol*. Mar 10 2011;29(8):994-1000. doi:10.1200/jco.2010.29.8315
21. Klinkhammer-Schalke M, Koller M, Steinger B, et al. Direct improvement of quality of life using a tailored quality of life diagnosis and therapy pathway: randomised trial in 200 women with breast cancer. *Br J Cancer*. Feb 28 2012;106(5):826-38. doi:10.1038/bjc.2012.4
22. Nicklasson M, Elfström ML, Olofson J, Bergman B. The impact of individual quality of life assessment on psychosocial attention in patients with chest malignancies: a randomized study. *Support Care Cancer*. Jan 2013;21(1):87-95. doi:10.1007/s00520-012-1496-6
23. Young JM, Butow PN, Walsh J, et al. Multicenter randomized trial of centralized nurse-led telephone-based care coordination to improve outcomes after surgical resection for colorectal cancer: the CONNECT intervention. *J Clin Oncol*. Oct 1 2013;31(28):3585-91. doi:10.1200/jco.2012.48.1036
24. Snyder CF, Herman JM, White SM, et al. When using patient-reported outcomes in clinical practice, the measure matters: a randomized controlled trial. *J Oncol Pract*. Sep 2014;10(5):e299-306. doi:10.1200/jop.2014.001413
25. Wheelock AE, Bock MA, Martin EL, et al. SIS.NET: a randomized controlled trial evaluating a web-based system for symptom management after treatment of breast cancer. *Cancer*. Mar 15 2015;121(6):893-9. doi:10.1002/cncr.29088
26. Basch E, Deal AM, Kris MG, et al. Symptom Monitoring With Patient-Reported Outcomes During Routine Cancer Treatment: A Randomized Controlled Trial. *J Clin Oncol*. Feb 20 2016;34(6):557-65. doi:10.1200/jco.2015.63.0830
27. Basch E, Deal AM, Dueck AC, et al. Overall Survival Results of a Trial Assessing Patient-Reported Outcomes for Symptom Monitoring During Routine Cancer Treatment. *Jama*. Jul 11 2017;318(2):197-198. doi:10.1001/jama.2017.7156
28. Mooney KH, Beck SL, Wong B, et al. Automated home monitoring and management of patient-reported symptoms during chemotherapy: results of the symptom care at home RCT. *Cancer Med*. Mar 2017;6(3):537-546. doi:10.1002/cam4.1002
29. Geerse OP, Hoekstra-Weebers JE, Stokroos MH, et al. Structural distress screening and supportive care for patients with lung cancer on systemic therapy: A randomised controlled trial. *Eur J Cancer*. Feb 2017;72:37-45. doi:10.1016/j.ejca.2016.11.006
30. Mertz BG, Dunn-Henriksen AK, Kroman N, et al. The effects of individually tailored nurse navigation for patients with newly diagnosed breast cancer: a randomized pilot study. *Acta Oncol*. Dec 2017;56(12):1682-1689. doi:10.1080/0284186x.2017.1358462

© 2024 Balitsky AK et al. *JAMA Network Open.*

31. Paterson C, Primeau C, Nabi G. A pilot randomised controlled trial of a multimodal supportive care (ThriverCare) intervention for managing unmet supportive care needs in men with metastatic prostate cancer on hormonal treatment and their partner/caregivers. *Eur J Oncol Nurs*. Dec 2018;37:65-73. doi:10.1016/j.ejon.2018.10.007
32. Tolstrup LK, Bastholt L, Dieperink KB, Möller S, Zwisler A-D, Pappot H. The use of patient-reported outcomes to detect adverse events in metastatic melanoma patients receiving immunotherapy: a randomized controlled pilot trial. *Journal of Patient-Reported Outcomes*. 2020/10/30 2020;4(1):88. doi:10.1186/s41687-020-00255-0
33. Bryant AL, Coffman E, Phillips B, et al. Pilot randomized trial of an electronic symptom monitoring and reporting intervention for hospitalized adults undergoing hematopoietic stem cell transplantation. *Support Care Cancer*. Mar 2020;28(3):1223-1231. doi:10.1007/s00520-019-04932-9
34. Fjell M, Langius-Eklöf A, Nilsson M, Wengström Y, Sundberg K. Reduced symptom burden with the support of an interactive app during neoadjuvant chemotherapy for breast cancer - A randomized controlled trial. *Breast*. Jun 2020;51:85-93. doi:10.1016/j.breast.2020.03.004
35. Handa S, Okuyama H, Yamamoto H, Nakamura S, Kato Y. Effectiveness of a Smartphone Application as a Support Tool for Patients Undergoing Breast Cancer Chemotherapy: A Randomized Controlled Trial. *Clin Breast Cancer*. Jun 2020;20(3):201-208. doi:10.1016/j.clbc.2020.01.004
36. Hentschel L, Richter S, Kopp HG, et al. Quality of life and added value of a tailored palliative care intervention in patients with soft tissue sarcoma undergoing treatment with trabectedin: a multicentre, cluster-randomised trial within the German Interdisciplinary Sarcoma Group (GISG). *BMJ Open*. Aug 27 2020;10(8):e035546. doi:10.1136/bmjopen-2019-035546
37. Riis CL, Jensen PT, Bechmann T, Möller S, Coulter A, Steffensen KD. Satisfaction with care and adherence to treatment when using patient reported outcomes to individualize follow-up care for women with early breast cancer - a pilot randomized controlled trial. *Acta Oncol*. Apr 2020;59(4):444-452. doi:10.1080/0284186x.2020.1717604
38. Moore EM, King TA, Wood EM, et al. Patient-reported outcome measures in multiple myeloma: Real-time reporting to improve care (My-PROMPT) - a pilot randomized controlled trial. *Am J Hematol*. Jul 2020;95(7):E178-e181. doi:10.1002/ajh.25815
39. Rogers SN, Allmark C, Bekiroglu F, et al. Improving quality of life through the routine use of the patient concerns inventory for head and neck cancer patients: baseline results in a cluster preference randomised controlled trial. *Eur Arch Otorhinolaryngol*. Dec 2020;277(12):3435-3447. doi:10.1007/s00405-020-06077-6
40. Klinkhammer-Schalke M, Steinger B, Koller M, et al. Diagnosing deficits in quality of life and providing tailored therapeutic options: Results of a randomised trial in 220 patients with colorectal cancer. *Eur J Cancer*. May 2020;130:102-113. doi:10.1016/j.ejca.2020.01.025
41. Rodin G, Malfitano C, Rydall A, et al. Emotion And Symptom-focused Engagement (EASE): a randomized phase II trial of an integrated psychological and palliative care intervention for patients with acute leukemia. *Support Care Cancer*. Jan 2020;28(1):163-176. doi:10.1007/s00520-019-04723-2
42. Absolom K, Warrington L, Hudson E, et al. Phase III Randomized Controlled Trial of eRAPID: eHealth Intervention During Chemotherapy. *J Clin Oncol*. Mar 1 2021;39(7):734-747. doi:10.1200/jco.20.02015
43. Judge MKM, Luedke R, Dyal BW, Ezenwa MO, Wilkie DJ. Clinical efficacy and implementation issues of an electronic pain reporting device among outpatients with cancer. *Support Care Cancer*. Sep 2021;29(9):5227-5235. doi:10.1007/s00520-021-06075-2
44. Pappot H, Baeksted CW, Nissen A, et al. Clinical effects of assessing electronic patient-reported outcomes monitoring symptomatic toxicities during breast cancer therapy: a nationwide and population-based study. *Breast Cancer*. Sep 2021;28(5):1096-1099. doi:10.1007/s12282-021-01244-x

© 2024 Balitsky AK et al. *JAMA Network Open.*

45. Riis CL, Stie M, Bechmann T, et al. ePRO-based individual follow-up care for women treated for early breast cancer: impact on service use and workflows. *J Cancer Surviv*. Aug 2021;15(4):485-496. doi:10.1007/s11764-020-00942-3
46. Warsame R, Cook J, Fruth B, et al. A prospective, randomized trial of patient-reported outcome measures to drive management decisions in hematology and oncology. *Contemp Clin Trials Commun*. Oct 2022;29:100964. doi:10.1016/j.conctc.2022.100964
47. Basch E, Schrag D, Henson S, et al. Effect of Electronic Symptom Monitoring on Patient-Reported Outcomes Among Patients With Metastatic Cancer: A Randomized Clinical Trial. *Jama*. Jun 28 2022;327(24):2413-2422. doi:10.1001/jama.2022.9265
48. Zhang L, Zhang X, Shen L, Zhu D, Ma S, Cong L. Efficiency of Electronic Health Record Assessment of Patient-Reported Outcomes After Cancer Immunotherapy: A Randomized Clinical Trial. *JAMA Network Open*. 2022;5(3):e224427-e224427. doi:10.1001/jamanetworkopen.2022.4427
49. Tolstrup LK, Pappot H, Bastholt L, Möller S, Dieperink KB. Impact of patient-reported outcomes on symptom monitoring during treatment with checkpoint inhibitors: health-related quality of life among melanoma patients in a randomized controlled trial. *J Patient Rep Outcomes*. Jan 21 2022;6(1):8. doi:10.1186/s41687-022-00414-5
50. Musoro JZ, Coens C, Sprangers MAG, et al. Minimally important differences for interpreting EORTC QLQ-C30 change scores over time: A synthesis across 21 clinical trials involving nine different cancer types. *European Journal of Cancer*. 2023/07/01/ 2023;188:171-182. doi:https://doi.org/10.1016/j.ejca.2023.04.027
51. Pickard AS, Neary MP, Cella D. Estimation of minimally important differences in EQ-5D utility and VAS scores in cancer. *Health and Quality of Life Outcomes*. 2007/12/21 2007;5(1):70. doi:10.1186/1477-7525-5-70
52. Yost KJ, Eton DT. Combining distribution- and anchor-based approaches to determine minimally important differences: the FACIT experience. *Eval Health Prof*. Jun 2005;28(2):172-91. doi:10.1177/0163278705275340
53. Cella D, Eton DT, Fairclough DL, et al. What is a clinically meaningful change on the Functional Assessment of Cancer Therapy-Lung (FACT-L) Questionnaire? Results from Eastern Cooperative Oncology Group (ECOG) Study 5592. *J Clin Epidemiol*. Mar 2002;55(3):285-95. doi:10.1016/s0895-4356(01)00477-2
54. Askew RL, Xing Y, Palmer JL, Cella D, Moye LA, Cormier JN. Evaluating minimal important differences for the FACT-Melanoma quality of life questionnaire. *Value Health*. Nov-Dec 2009;12(8):1144-50. doi:10.1111/j.1524-4733.2009.00570.x
55. Locke DE, Decker PA, Sloan JA, et al. Validation of single-item linear analog scale assessment of quality of life in neuro-oncology patients. *J Pain Symptom Manage*. Dec 2007;34(6):628-38. doi:10.1016/j.jpainsymman.2007.01.016
56. Yost KJ, Cella D, Chawla A, et al. Minimally important differences were estimated for the Functional Assessment of Cancer Therapy-Colorectal (FACT-C) instrument using a combination of distribution- and anchor-based approaches. *J Clin Epidemiol*. Dec 2005;58(12):1241-51. doi:10.1016/j.jclinepi.2005.07.008

© 2024 Balitsky AK et al. *JAMA Network Open.*
