# Supplementary material for: Patient-Reported Outcome Measures in Cancer Care: An Updated Systematic Review and Meta-Analysis
Source: JAMA Netw Open. 2024 Aug 13;7(8):e2424793. doi: 10.1001/jamanetworkopen.2024.24793 (PMC11322847; doi:10.1001/jamanetworkopen.2024.24793)
Supplement: Supplement 2. — Data Sharing Statement [file jamanetwopen-e2424793-s002.pdf]

Data Sharing Statement

Balitsky. Patient-Reported Outcome Measures in Cancer Care. *JAMA Netw Open*. Published August 09, 2024. doi:10.1001/jamanetworkopen.2024.24793

Data
Data available: No

Additional Information
Explanation for why data not available: We performed a systematic review and therefore do not have patient-specific data
